# HSP60 knockdown exerts differential response in endothelial cells and monocyte derived macrophages during atherogenic transformation

**DOI:** 10.1038/s41598-020-79927-2

**Published:** 2021-01-13

**Authors:** Kavita Shirsath, Apeksha Joshi, Aliasgar Vohra, Ranjitsinh Devkar

**Affiliations:** Department of Zoology, Faculty of Science, The Maharaja Sayajirao University of Baroda, Vadodara, 390002 Gujarat India

**Keywords:** Atherosclerosis, Mechanisms of disease

## Abstract

Ectopic expression of HSP60 in vascular cells is known to activate auto-immune response that is critical to atherogenic initiation. However, the pathogenic relevance of the aberrant HSP60 upregulation in intracellular signaling pathways associated with atherogenic consequences in vascular cells remains unclear. The aim of the present study was to determine the role of endogenous HSP60 in atherogenic transformation of endothelial cells and macrophages. After generating primary evidence of oxidized low density lipoprotein (OxLDL) induced HSP60 upregulation in human umbilical vein endothelial cells (HUVEC), its physiological relevance in high fat high fructose (HFHF) induced early atherogenic remodelling was investigated in C57BL/6J mice. Prominent HSP60 expression was recorded in tunica intima and media of thoracic aorta that showed hypertrophy, lumen dilation, elastin fragmentation and collagen deposition. Further, HSP60 overexpression was found to be prerequisite for its surface localization and secretion in HUVEC. eNOS downregulation and MCP-1, VCAM-1 and ICAM-1 upregulation with subsequent macrophage accumulation provided compelling evidences on HFHF induced endothelial dysfunction and activation that were also observed in OxLDL treated- and HSP60 overexpressing-HUVEC. OxLDL induced concomitant reduction in NO production and monocyte adhesion were prevented by HSP60 knockdown, implying towards HSP60 mediated possible regulation of the said genes. OxLDL induced HSP60 upregulation and secretion was also recorded in THP-1 derived macrophages (TDMs). HSP60 knockdown in TDMs accounted for higher OxLDL accumulation that correlated with altered scavenger receptors (SR-A1, CD36 and SR-B1) expression further culminating in M1 polarization. Collectively, the results highlight HSP60 upregulation as a critical vascular alteration that exerts differential regulatory role in atherogenic transformation of endothelial cells and macrophages.

## Introduction

Development of early atherosclerotic lesion is preceded by endothelial cell (EC) dysfunction involving impairment of nitric oxide (NO)-mediated vaso-relaxation. These changes in EC are accompanied by loss of barrier function allowing the sub-endothelial accumulation of low density lipoprotein (LDL). Simultaneous expression of adhesion molecules including intercellular cell adhesion molecule-1 (ICAM-1), vascular cell adhesion molecule-1 (VCAM-1) and E-selectin on EC cell surface along with secretion of chemokines like macrophage chemoattractant protein-1 (MCP-1), facilitates sub-intimal recruitment of macrophages that marks the initiation of early atherosclerotic lesions. The resultant inflammation mediates vascular remodelling that gradually culminates in development of atheromatous plaques^1,2^. Thus, EC dysfunction is a critical event that triggers the development of atherosclerotic lesions and it is imperative to understand the underlying molecular mechanisms.

The development of mature atheromatous plaque primarily involves internalization of oxidized LDL (OxLDL) by macrophages and their transformation into foam cells^3^. The uptake of OxLDL via scavenger receptors (SRs), such as scavenger receptor class A type 1 (SR-A1) and cluster of differentiation 36 (CD36), and subsequent receptor-mediated endocytosis activates a cascade of pathogenic events involving mitochondrial depolarization, generation of reactive oxygen species (ROS) as well as upregulation of SRs^4^. Apart from uptake, the degree of lipid accumulation is also affected by cholesterol efflux mechanisms involving scavenger receptor class B type 1 (SR-B1)^5^. However, the mechanisms governing the expression of SRs and subsequent foam cell formation are rather complex and require clarification. Though, the role of variety of proteins and signaling molecules have been deciphered, molecular chaperones such as heat shock protein 60 (HSP60), HSP70, HSP90 and HSP27 have been implied in plaque formation with variable degrees of severity^6–9^. Amongst others, HSP60 has been of particular interest because its prominent expression has been strongly linked with early stages of the disease development^10^, that can be potentially reversed by therapeutic interventions.

HSP60 is a molecular chaperone that predominantly functions in mitochondrial protein folding and is upregulated in conditions of mitochondrial stress^11^. The primary evidence in support of pro-atherogenic association of HSP60 came from epidemiological studies showing accumulation of HSP60-reactive T-cells in early arterial lesions^10,12^. In this context, ectopic expression of HSP60 in ECs exposed to classical atherogenic risk factors have been demonstrated^13–17^. The co-expression of adhesion molecules and HSP60 in stressed endothelium plays a crucial role in infiltration of HSP60 cross-reactive mononuclear cells and subsequent inflammation of vascular wall^12,16^. Besides, circulating levels of HSP60 and anti-HSP60 antibodies have also been correlated with severity of atheromatous plaques in human and murine models^9,18^ that supports the auto-immune concept of atherosclerosis. Moreover, soluble HSP60 in the extracellular milieu is also known to activate inflammatory immune response in macrophages via toll-like receptor 4 (TLR-4) activation^19^, thus contributing towards atherogenic progression. Activation of adaptive immune responses during advanced stages of plaques have also been attributed to the soluble HSP60^20^. Thus, the auto-antigenic role of HSP60 in early stages of atherosclerosis is known, but the significance of HSP60 upregulation in stressed ECs during atherogenic initiation remains unexplored.

Several studies have reported cytosolic presence of HSP60 and drawn correlations with its role in intracellular signaling pathways. In cancer cells, cytosolic HSP60 had been observed to interact with inhibitor of κB kinase (IKK) complex for activation of NF-κB pathway^21^. Further, HSP60 mediated regulation of apoptotic pathway has also been shown in various types of cells^22,23^. Overexpression of HSP60 has been reported to induce mitogenic actions in vascular smooth muscle cells (VSMCs) that emphasize the intracellular functions of HSP60 in vascular cells^24^. Since stress induced overexpression of HSP60 in ECs precede its cell surface translocation^14^ and secretion^25^, it can be believed to be involved in regulation of intracellular signaling pathways associated with related atherogenic events. Also, the surface localization of HSP60 in vascular endothelium is known^14,15^ but, OxLDL mediated atherogenic events governed by HSP60 have not been studied. HSP60 upregulation induced by OxLDL has been observed in monocytic cell lines^26^ emphasizing a cause-and-effect relationship between OxLDL and HSP60 during atherogenic transformation of macrophages that also needs a detailed scrutiny. The present study aims at exploring the modulations in HSP60 expression and its role in regulation of OxLDL induced atherogenic changes in EC and macrophages.

First, OxLDL mediated changes in HSP60 expression was evaluated in human umbilical vein endothelial cells (HUVEC), followed by assessment of HSP60 status during early atherogenic remodelling of thoracic aorta induced by high fat high fructose (HFHF) diet in C57BL/6J mice. Correlations of HSP60 upregulation with endothelial dysfunction and activation were drawn and the underlying mechanism was investigated by silencing HSP60 expression in HUVEC. OxLDL induced endothelial dysfunction and activation were reduced in conditions of HSP60 knockdown in HUVEC. Further, the expression of HSP60 was also assessed in OxLDL mediated foam cell formation in THP-1 derived macrophages (TDMs). The modulatory role of HSP60 on accumulation of OxLDL in TDMs via SRs and its impact on macrophage polarization was assessed by silencing HSP60. Lowered levels of HSP60 was found to alter the SRs expression so as to favour higher OxLDL accumulation and subsequent polarization to M1 subtype.

## Results

### OxLDL upregulates endogenous HSP60 in HUVEC

**Figure 1:**
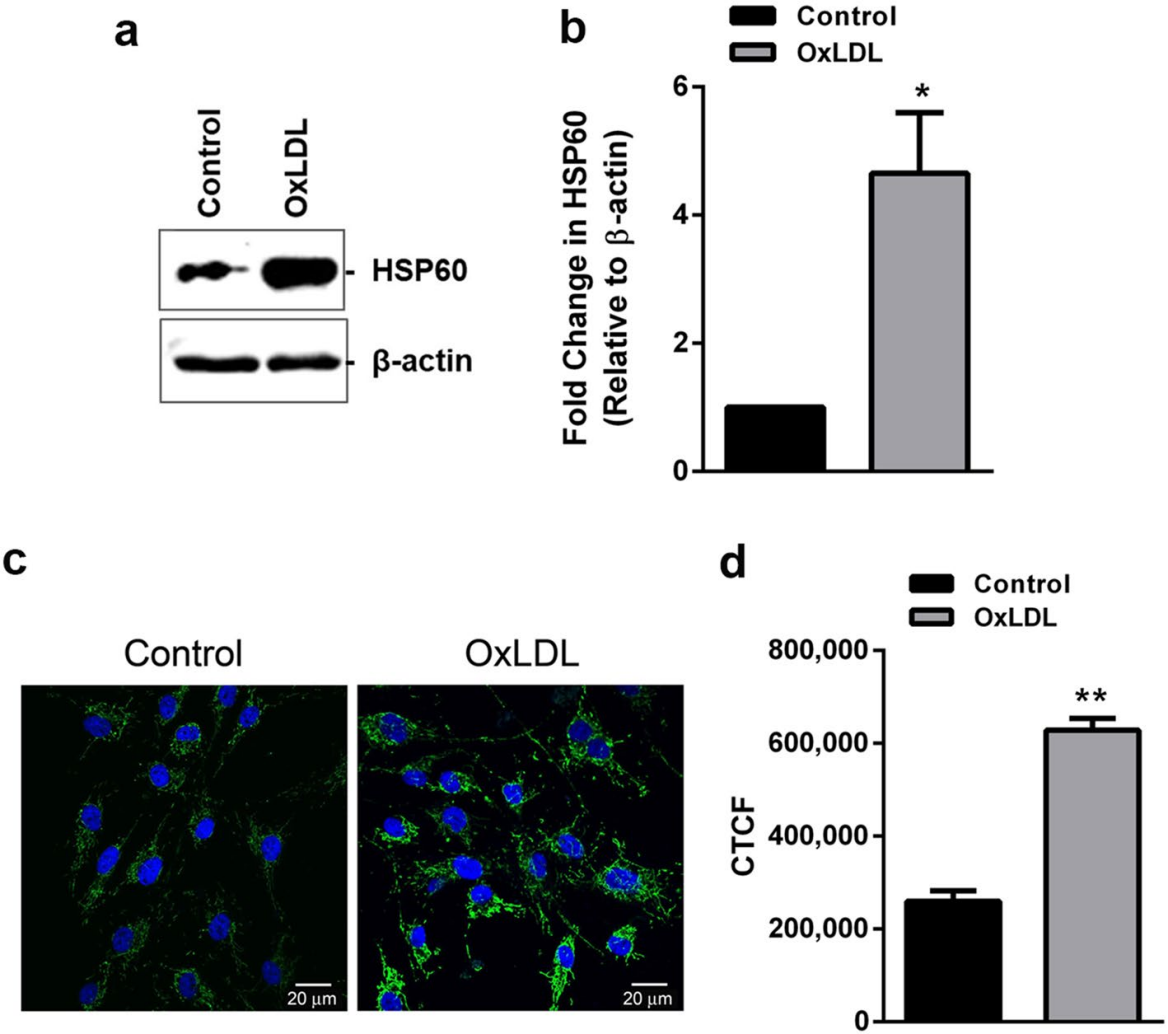
HSP60 expression in OxLDL treated HUVEC. Cells were treated with OxLDL (80 µg/ml) for 24 h and expression of HSP60 was evaluated by (**a**) western blotting followed by (**b**) its densitometric analysis and (**c**) immunocytochemistry followed by (**d**) its quantification by Image J analysis. Scale bar = 20 µm. *CTCF* corrected total cell fluorescence. Data were expressed as Mean ± SEM (n = 3). *p < 0.05, **p < 0.01 vs untreated control, unpaired two-tailed Student’s t-test.

HUVEC stimulated with OxLDL (80 µg/ml for 24 h) accounted for ~ 26% cell death (p < 0.05) as compared to control (Supplemental Fig. S1) and a significant upregulation of HSP60 as evidenced by its mRNA (p < 0.001; Supplemental Fig. S2) and protein expression (p < 0.05; Fig. 1a,b). Further confirmation in this regard was obtained by immunostaining of HSP60 wherein, OxLDL treated HUVEC showed significantly higher immunofluorescence (p < 0.01) as compared to control cells (Fig. 1c,d).

### HFHF diet induces vascular remodelling and HSP60 upregulation in thoracic aorta

**Figure 2:**
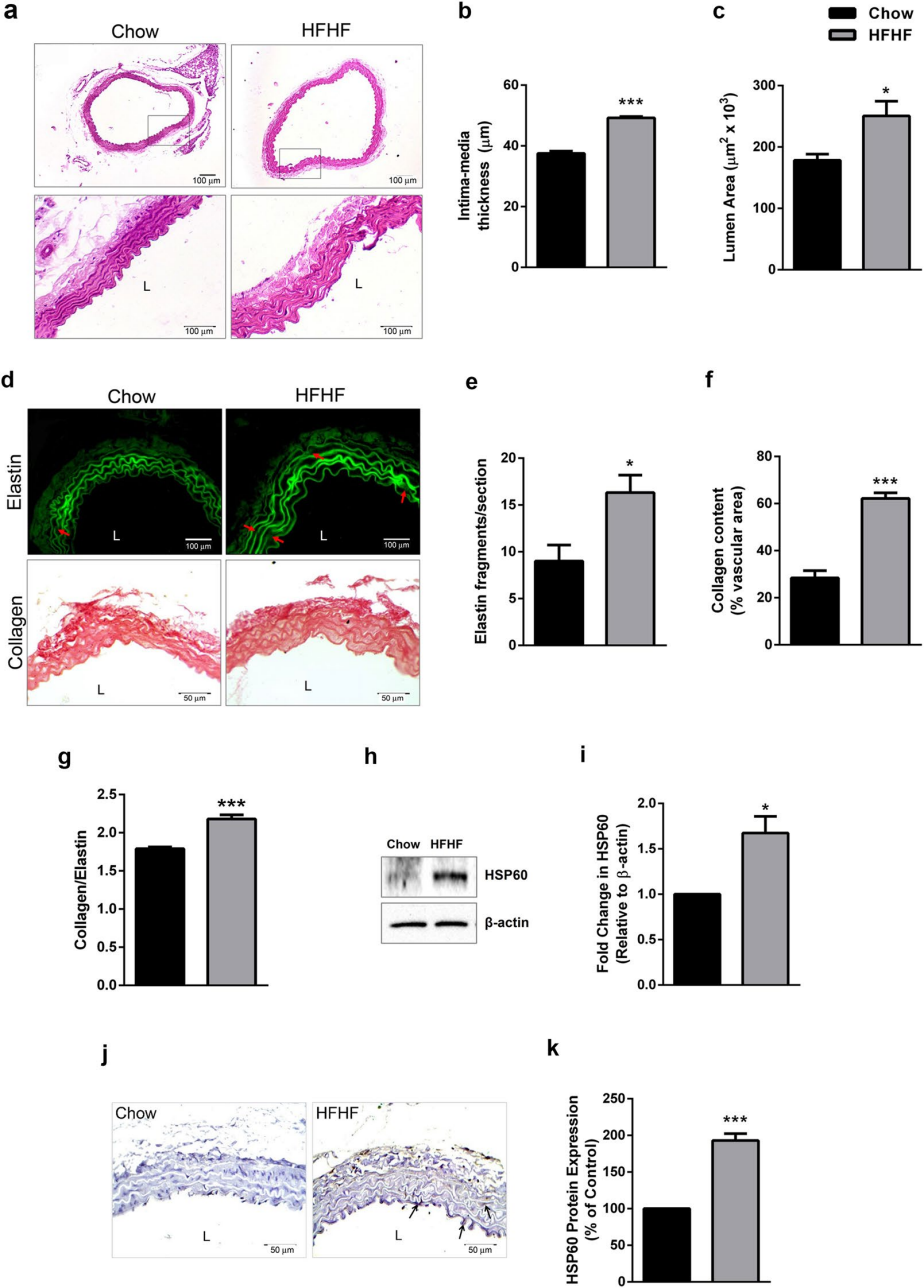
Pro-atherogenic remodelling and HSP60 expression in thoracic aorta of HFHF diet fed mice. (**a**) Histological analysis of thoracic aortas stained with H × E (100× and 400×) were subjected to quantification of (**b**) Intima-media thickness (IMT) (n = 3; Scale bar = 100 µm) and (**c**) lumen area (n = 3). The sections were also subjected to (**d**) elastin autofluorescence analysis (upper lane, red arrows indicate elastin breaks; Scale bar = 100 µm) and collagen staining by picrosirius red (lower lane; Scale bar = 50 µm). The graphs represents (**e**) elastin fragmentation (n = 3), (**f**) collagen content (n = 5 for control, n = 4 for HFHF) and (**g**) collagen-to-elastin ratio (n = 4). The expression of HSP60 was analysed by (**h**) western blotting followed by (**i**) densitometry (n = 3) and (**j**) immunohistochemistry (Scale bar = 50 µm; arrows indicate HSP60^+^ stained areas) followed by (**k**) quantification (n = 3) by Image J. Data were expressed as Mean ± SEM. *p < 0.05, ***p < 0.001 vs Chow diet fed mice, unpaired two-tailed Student’s t-test. *L* lumen.

Thoracic aorta of C57BL/6J mice fed with HFHF diet (for 16 weeks) was investigated for possible changes in HSP60 after confirming early atherogenic changes. HFHF diet fed mice showed significantly higher weight gain (p < 0.001; Supplemental Fig. S3a) but, food intake was lower as compared to control (p < 0.001; Supplemental Fig. S3b). The same was attributable to higher calorie value of high fat diet as compared to Chow diet (Supplemental Table S1). Further, feeding of HFHF diet accounted for significant increment in triglycerides (p < 0.05), total cholesterol (p < 0.01), VLDL (p < 0.001), LDL-cholesterol (p < 0.05) and decrement in HDL-cholesterol (p < 0.05) resulting in higher (p < 0.01) LDL-Chol/HDl-Chol ratio (Supplemental Fig. S4a–f). Microscopic evaluation of thoracic aorta (H × E stained; 100 × and 400 ×) showed a significant increment in intima-media thickness (p < 0.001) and lumen area (p < 0.05) following HFHF feeding (Fig. 2a–c). Evidence on HFHF mediated elastin derangement was obtained in autofluorescent sections of thoracic aorta (Fig. 2d upper lane) that showed significantly higher breaks (p < 0.05) as compared to control (Fig. 2e). Picrosirius red stained section of thoracic aorta of HFHF diet fed mice showed thickening in the outer collagen area (Fig. 2d lower lane), higher collagen deposition (p < 0.001; Fig. 2f) and higher collagen: elastin ratio (p < 0.001; Fig. 2g).

Thoracic aorta of HFHF diet fed mice recorded a significantly higher expression of HSP60 protein (p < 0.05; Fig. 2h,i) and prominent immunostaining of HSP60 (p < 0.001) in tunica media and intima (Fig. 2j,k).

### Surface localization and secretion of HSP60 in HUVEC

**Figure 3:**
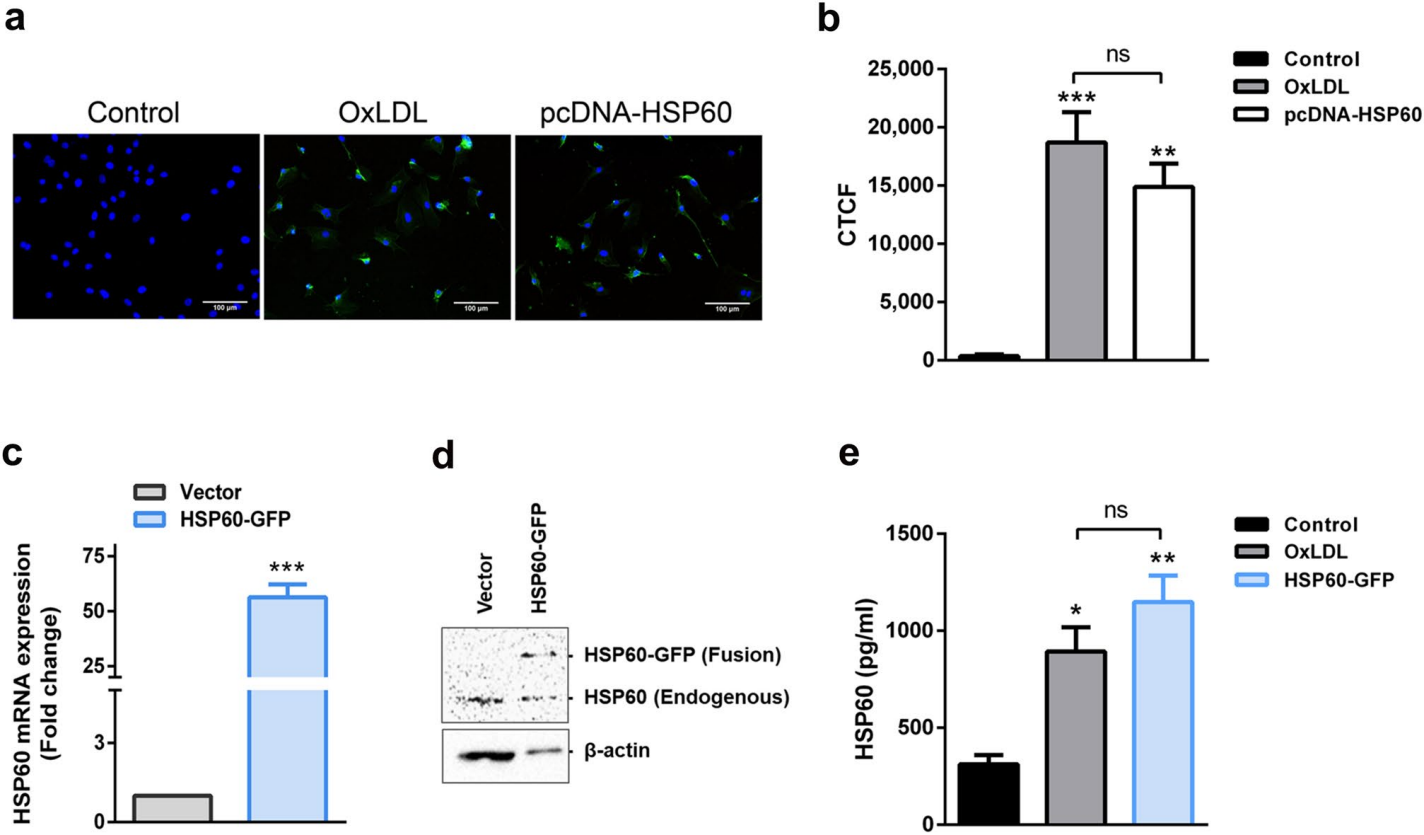
Surface localization and secretion of HSP60 in HUVEC. (**a**) OxLDL treated and pcDNA-HSP60 HUVECs were surface immunostained for HSP60 by following 1% PFA fixation protocol. Representative images are shown with HSP60 (A488), β-actin (A568) and nuclei (Hoechst). Scale bar = 100 µm. (**b**) The fluorescence was quantified using Image J. *CTCF* corrected total cell fluorescence (n = 4 for control, n = 3 for OxLDL and pcDNA-HSP60). **p < 0.01, ***p < 0.001 vs untreated control, One way ANOVA followed by Tukey’s multiple comparison test. Further, the transient overexpression of HSP60 in HSP60-GFP cells was assessed by (**c**) quantitative RT-PCR and (**d**) western blotting (n = 3). ***p < 0.001 vs Vector, unpaired two-tailed Student’s t-test. (**e**) The levels of HSP60 in conditioned media from HUVEC were analysed by ELISA (n = 3). Data were represented as Mean ± SEM. *p < 0.05, **p < 0.01 vs untreated control, *ns* non-significant, One way ANOVA followed by Tukey’s multiple comparison test.

For surface localization study, overexpression of HSP60 in HUVEC was achieved using pcDNA-HSP60 and the same was confirmed by its immunostaining (Supplemental Fig. S5a). Further, immunocytochemical staining of plasma membrane localized-HSP60 was standardized by co-staining of HSP60 and β-actin^14^, the latter serving as an internal positive and negative controls for intracellular and surface staining, respectively. We observed that 1% PFA fixation ensured plasma membrane integrity that allowed surface detection of HSP60 with β-actin remaining unstained (Supplemental Fig. S5b upper lane). 2% PFA + MeOH fixation led to cell membrane permeabilization leading to intracellular dual staining of HSP60 and β-actin (Supplemental Fig. S5b lower lane). Further, surface localization of HSP60 was checked in OxLDL treated HUVEC and pcDNA-HSP60 groups that recorded prominent fluorescence of HSP60 on cell membrane as compared to control (p < 0.001, p < 0.01, respectively; Fig. 3a,b). Vector cells that were transfected with empty plasmid vector, did not record any fluorescence on plasma membrane similar to control cells (Supplemental Fig. S6).

Evidence on HSP60 secretion was obtained by carrying out ELISA of the conditioned media. Significant upregulation of HSP60 mRNA (p < 0.001) and protein (Fig. 3c,d) in HSP60-GFP cells confirmed its overexpression without notable cell death (Supplemental Fig. S7). Further, OxLDL treatment was instrumental in facilitating significantly higher HSP60 secretion (p < 0.05) as compared to control. Also, HSP60-GFP cells recorded a significant increment (p < 0.01) in HSP60 secretion that was also in keeping with its overexpression (Fig. 3e). At this stage, LDH assay for OxLDL treated and HSP60-GFP cells were performed and the results showed no significant LDH release in both these groups as compared to their respective controls (Supplemental Fig. S8a,b) confirming that the detected HSP60 is indeed a secretory product and not a consequence of cell lysis.

### Validation of HFHF diet induced endothelial dysfunction and activation in thoracic aorta

**Figure 4:**
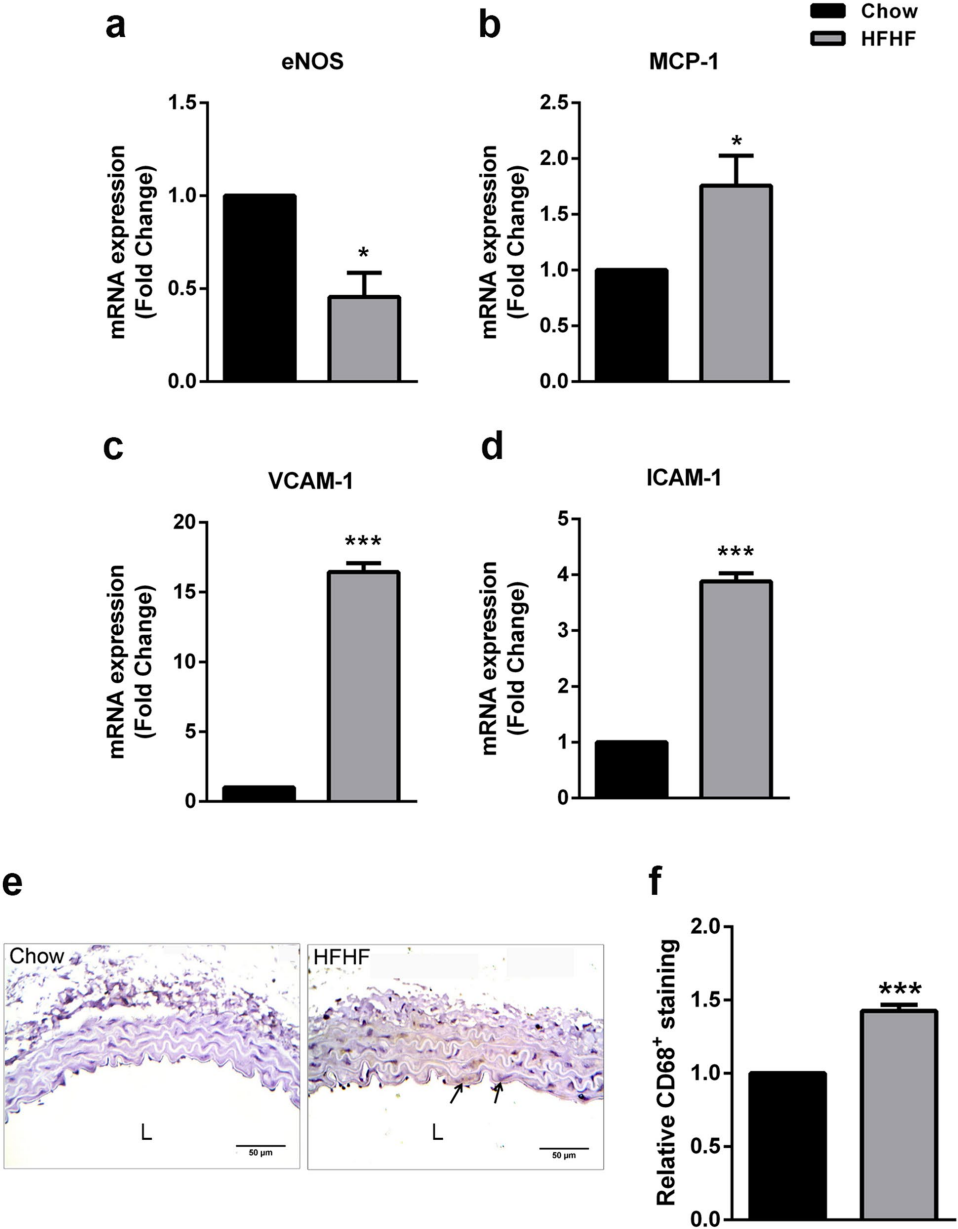
Endothelial dysfunction and activation in thoracic aorta of HFHF diet fed mice. Thoracic aorta from Control and HFHF groups were subjected to quantitative RT-PCR for (**a**) eNOS, (**b**) MCP-1, (**c**) VCAM-1 and (**d**) ICAM-1 (n = 3). (**e**) Sections were immunostained for CD68 (Scale bar = 50 µm; arrows indicate CD68^+^ staining). (**f**) Quantification of the positively stained areas was carried out using Image J (n = 5). Data were expressed as Mean ± SEM. *p < 0.05, **p < 0.01, ***p < 0.001 vs untreated control, unpaired two-tailed Student’s t-test. *L* lumen.

Endothelial nitric oxide synthase (eNOS) was assessed in thoracic aorta of HFHF fed mice, as NO released by vascular endothelium mediates vaso-relaxation^1^. In our study, eNOS mRNA was significantly lowered (p < 0.05) in thoracic aorta of HFHF group as compared to control (Fig. 4a) implying towards endothelial dysfunction. Macrophage chemoattractant protein 1 (MCP-1) mediated recruitment of monocytes in sub-intimal space coupled with upregulation of vascular cell adhesion molecule 1 (VCAM-1) and intercellular adhesion molecule 1 (ICAM-1) are key atherogenic changes that were recorded herein, in form of significantly higher mRNA levels of the said indicators (p < 0.05, p < 0.001, p < 0.001, respectively) in HFHF fed mice (Fig. 4b–d). Presence of significantly higher (p < 0.01) CD68 (macrophage marker) positive area were recorded in HFHF fed mice as compared to control (Fig. 4e,f).

### HSP60 regulates endothelial dysfunction in OxLDL treated HUVEC

**Figure 5:**
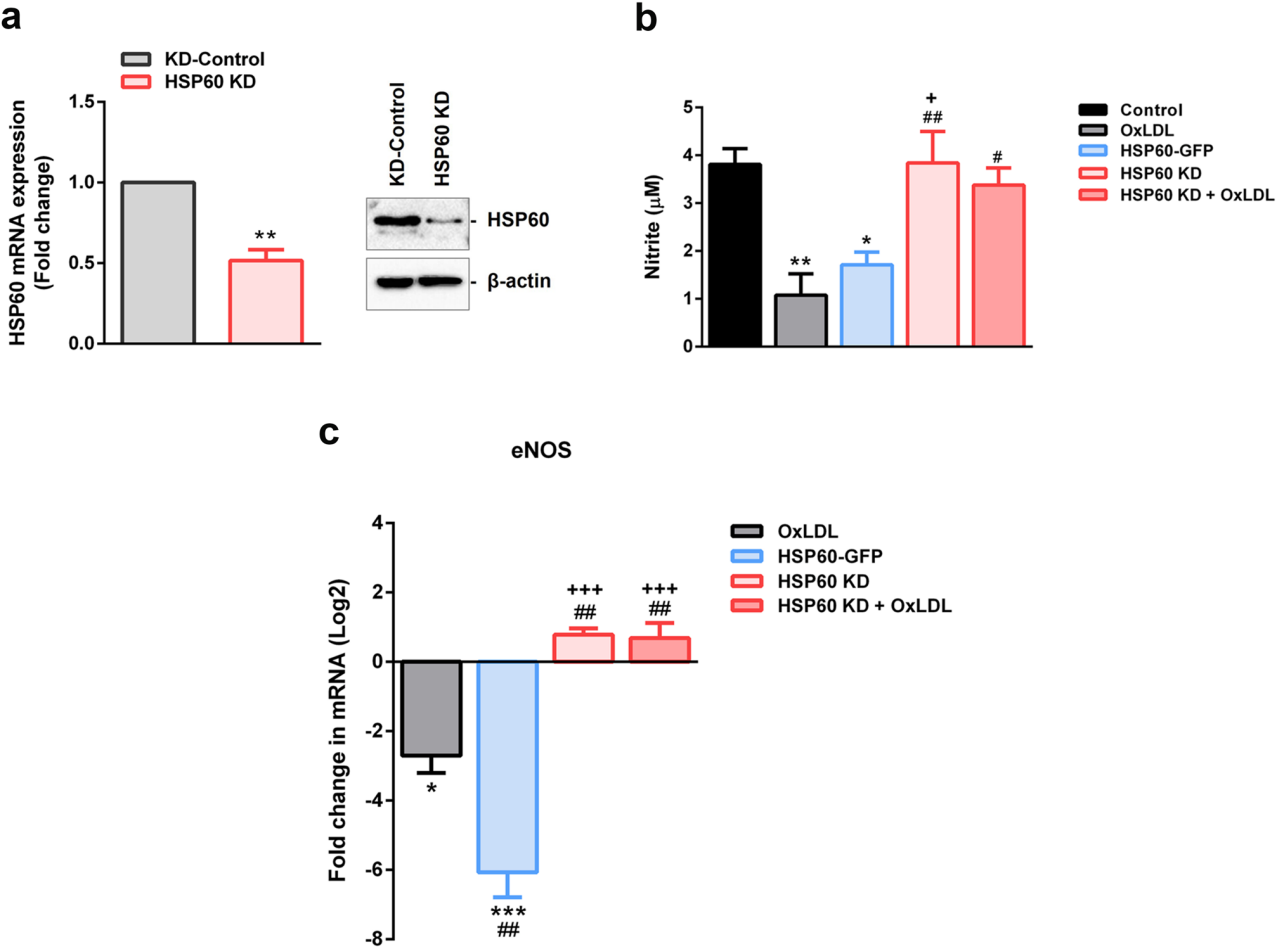
HSP60 mediated endothelial dysfunction in HUVEC. (**a**) Knockdown of HSP60 in HUVEC was confirmed by quantitative RT-PCR and western blotting. *p < 0.05, **p < 0.01, ***p < 0.001 vs KD-control, unpaired two-tailed Student’s t-test. (**b**) NO production from HUVEC was assessed using Griess’ reagent and (**c**) expression of eNOS mRNA was analysed by quantitative RT-PCR. Data were expressed as Mean ± SEM (n = 3). *p < 0.05, **p < 0.01, ***p < 0.001 vs untreated control, ^#^p < 0.05, ^##^p < 0.01, ^###^p < 0.001 vs OxLDL, and ^+^p < 0.05, ^+++^p < 0.001 vs HSP60-GFP, One way-ANOVA followed by Tukey’s multiple comparison test.

Relevance of HSP60 in vascular endothelium and atherogenic condition was studied in OxLDL treated HUVEC wherein HSP60 status was experimentally altered. HSP60 KD cells (knockdown) recorded significant decrement (p < 0.01) in HSP60 mRNA and protein levels as compared to KD-Control cells (Fig. 5a, Supplemental Fig. S9a). HSP60 KD cells also recorded ~ 21% cell death (p < 0.01) that was further enhanced to ~ 42% (p < 0.001) overall cell mortality following OxLDL treatment (Supplemental Fig. S9b).

Markers of endothelial dysfunction (eNOS and NO) were assessed in OxLDL, HSP60-GFP, HSP60 KD, HSP60 KD + OxLDL groups. The total NO production was assessed by Griess’ reagent wherein, the results showed significant decrement in the nitrite content in OxLDL treated (p < 0.01) and HSP60-GFP groups (p < 0.05). However, HSP60 knockdown prevented the said decrement as the NO levels in HSP60 KD and HSP60 KD + OxLDL groups were comparable to control (Fig. 5b). mRNA levels of eNOS were downregulated in OxLDL treated (p < 0.05) and HSP60-GFP cells (p < 0.001). However, moderate increment (non-significant) was recorded in HSP60 KD and HSP60 KD + OxLDL groups (Fig. 5c). These findings imply towards lack of OxLDL mediated lowering of eNOS in conditions of HSP60 knockdown.

### HSP60 regulates endothelial activation in OxLDL treated HUVEC

**Figure 6:**
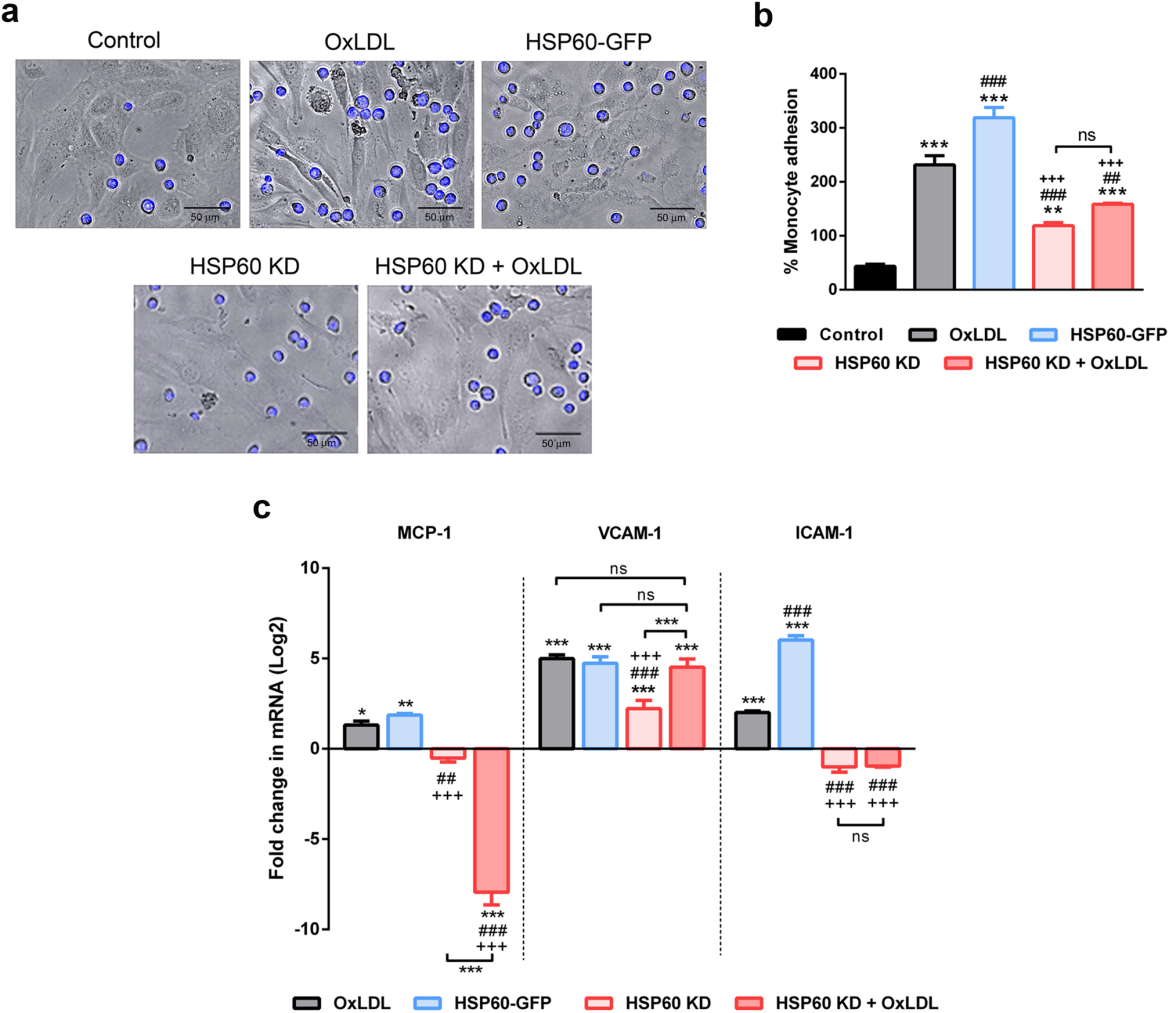
HSP60 mediated endothelial activation in HUVEC. The adhesion of THP-1 cells to HUVEC subjected to various experimental conditions was assessed by (**a**) microscopic analysis and (**b**) the % monocyte adhesion was calculated relative to number of HUVECs (n = 5). Scale bar = 50 µm. **p < 0.01, ***p < 0.001 vs untreated control, ^##^p < 0.01, ^###^p < 0.001 vs OxLDL, and ^+++^p < 0.001 vs HSP60-GFP, One way-ANOVA followed by Tukey’s multiple comparison test. (**c**) mRNA of MCP-1, VCAM-1 and ICAM-1 was assessed by quantitative RT-PCR (n = 3). Data were expressed as Mean ± SEM. *p < 0.05, **p < 0.01, ***p < 0.001 vs untreated control, ^##^p < 0.01, ^###^p < 0.001 vs OxLDL, and ^+++^p < 0.001 vs HSP60-GFP, Two way-ANOVA followed by Tukey’s multiple comparison test.

Adhesion assay using THP-1 cells was studied in OxLDL, HSP60-GFP, HSP60 KD and HSP60 KD + OxLDL HUVEC to assess HSP60 mediated endothelial activation. Results showed that OxLDL treated cells had more number of adhering monocytes (p < 0.001) compared to control. The HSP60-GFP group showed significantly higher (p < 0.001) % monocyte adhesion whereas HSP60 KD groups accounted for significantly lower numbers of adhering monocytes (p < 0.001, p < 0.001, respectively) (Fig. 6a,b). The endothelial activation markers (MCP-1, VCAM-1 and ICAM-1) were upregulated by OxLDL treatment as evidenced by their mRNA levels (p < 0.05, p < 0.001, p < 0.001, respectively). The increment in mRNA levels was more prominent (p < 0.01, p < 0.001, p < 0.001, respectively) HSP60-GFP group. HSP60 KD group recorded significant increment in VCAM-1 mRNA (p < 0.001) but, the value was significantly lower than OxLDL treated cells (p < 0.001) and HSP60-GFP cells (p < 0.001). MCP-1 and ICAM-1 mRNAs recorded non-significant decrement in HSP60 KD cells. OxLDL treatment to HSP60 KD cells recorded significant decrement in MCP-1 (p < 0.001) and significant increment in VCAM-1 mRNA (p < 0.001). ICAM-1 mRNA of this group showed a non-significant decrement (Fig. 6c).

### OxLDL induces upregulation and secretion of HSP60 in TDMs

**Figure 7:**
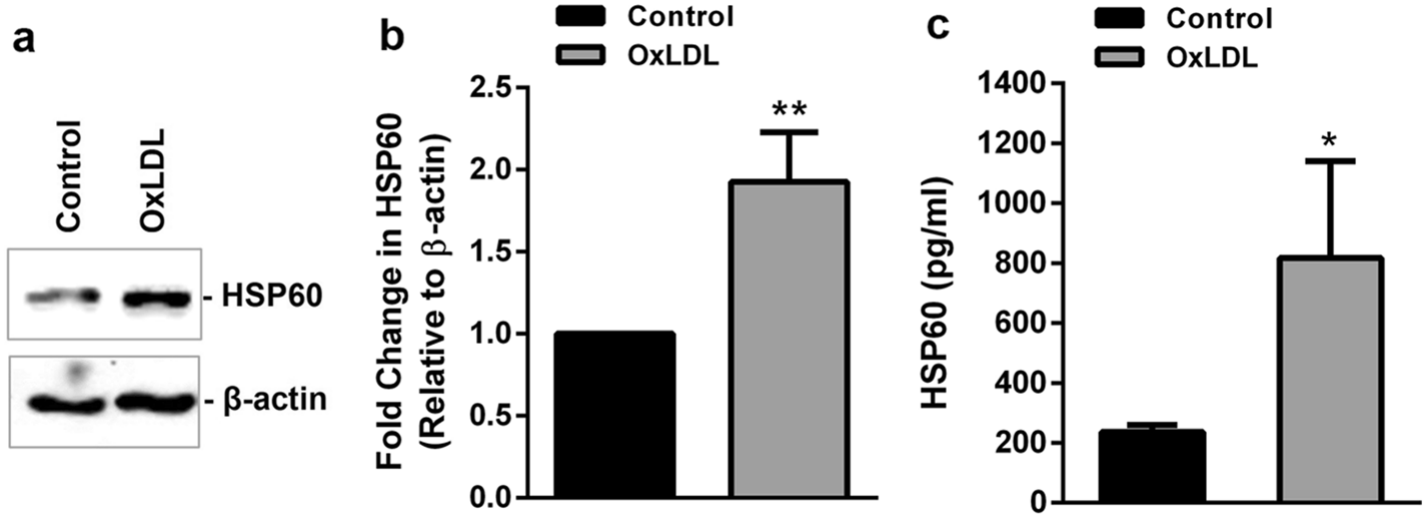
Expression and secretion of HSP60 in OxLDL treated TDMs. TDMs were treated with OxLDL and expression of HSP60 was evaluated by (**a**) western blotting followed by (**b**) densitometry. (**c**) HSP60 secretion was checked in conditioned media by ELISA. Data were expressed as Mean ± SEM (n = 3). *p < 0.05, **p < 0.01, vs untreated control, unpaired two-tailed Student’s t-test.

The results of MTT assay showed a significant cell death of ~ 29% in TDMs on treatment with OxLDL (80 µg/ml) as compared to control (Supplemental Fig. S10a). Further, significant upregulation of HSP60 protein was observed in presence of OxLDL (Fig. 7a,b). To determine the secretion of HSP60 under the influence of OxLDL, HSP60 levels in conditioned media of OxLDL treated TDMs was measured using anti-HSP60 ELISA. TDMs secreted significantly higher HSP60 on exposure to OxLDL (p < 0.05) compared to control (Fig. 7c). LDH assay did not record significant cell lysis in presence of OxLDL as compared to untreated TDMs (Supplemental Fig. S10b), indicating an active secretion of HSP60.

### HSP60 downregulation enhances OxLDL uptake in TDMs

**Figure 8:**
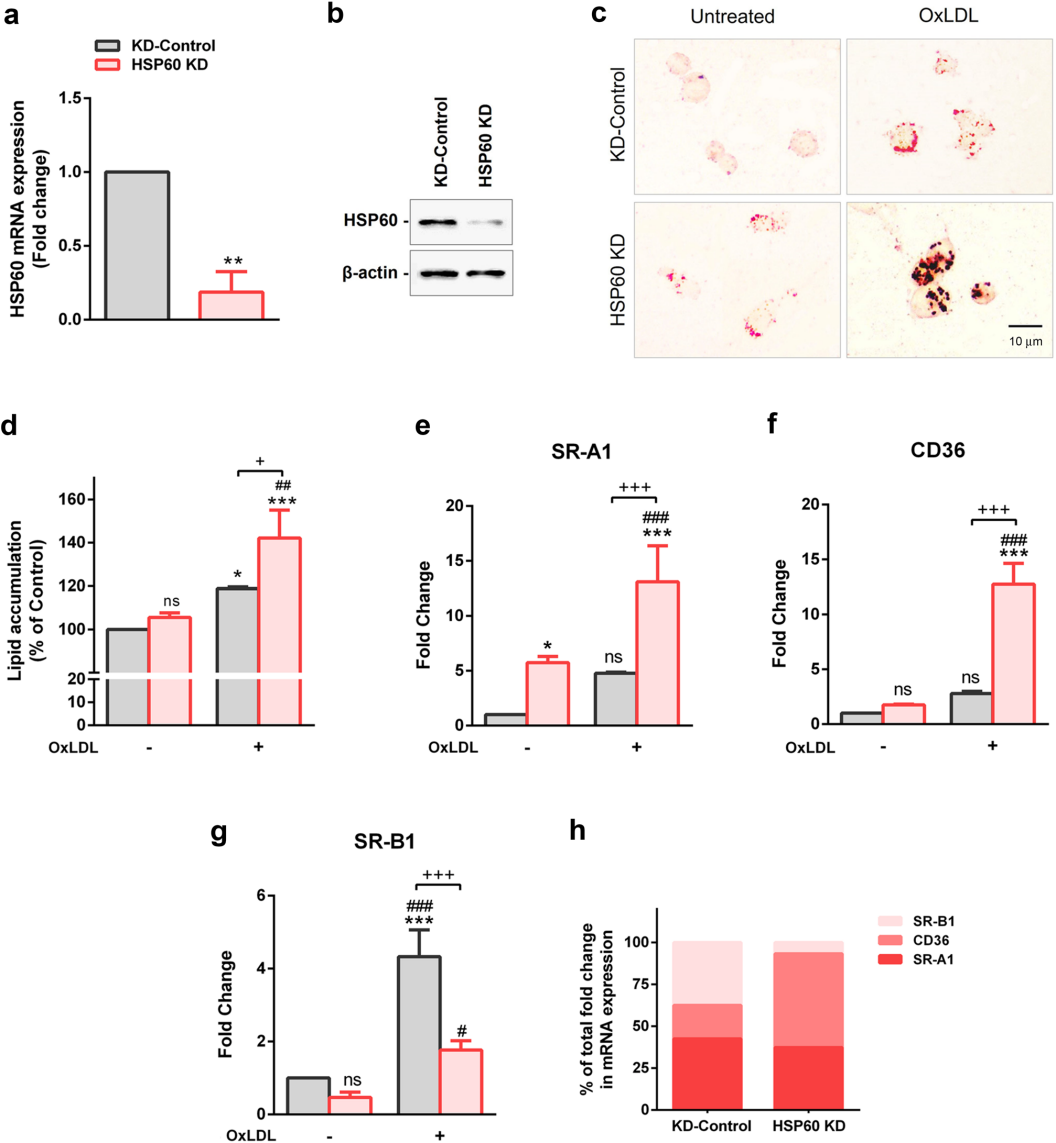
OxLDL uptake and scavenger receptor expression in HSP60 KD TDMs. Knockdown of HSP60 in TDMs was confirmed by (**a**) quantitative RT-PCR and (**b**) western blotting (n = 3). **p < 0.01, vs untreated KD-Control, two-tailed Student’s t-test. TDMs were subjected to ORO staining to check the OxLDL uptake (**c**). Representative images of stained cells (Scale bar = 10 µm) and (**d**) quantitative measurement of OxLDL accumulation. mRNA expression of (**e**) SR-A1, (**f**) CD36 and (**g**) SR-B1 was evaluated by quantitative RT-PCR. (**h**) Comparative analysis of mRNA expression of the three SRs in OxLDL treated KD-Control and HSP60 KD TDMs expressed as percentage of total fold change observed in respective groups. Data were expressed as Mean ± SEM (n = 3). *p < 0.05, ***p < 0.001 vs untreated KD-Control. ^#^p < 0.05, ^##^p < 0.01, ^###^p < 0.001 vs untreated HSP60 KD. ^+^p < 0.05, ^+++^p < 0.001 KD-Control group vs HSP60 KD group, *ns* non-significant, Two-way ANOVA followed by Tukey’s multiple comparison test.

Quantitative RT-PCR and western blot analysis showed successful knockdown of HSP60 (Fig. 8a,b, Supplemental Fig. S11a). However, the same caused a reduction in cell viability by ~ 24% (p < 0.05), which further recorded a non-significant decrement of ~ 12% on treatment with OxLDL (Supplemental Fig. S11b). Further, the accumulation OxLDL in TDMs was assessed by ORO staining. Both qualitative and quantitative results showed a significant increase in cytoplasmic lipid droplets in OxLDL treated TDMs (p < 0.05). Interestingly, exposure of OxLDL accounted for a significantly higher lipid accumulation (~ 42%) in HSP60 KD cells (p < 0.001) compared to that in KD-Control (Fig. 8c,d). To understand the augmented lipid accumulations in HSP60 KD cells, the mRNA expression of SRs were analysed. As shown in Fig. 8e, SR-A1 mRNA levels were found to be increased moderately (non-significant) in OxLDL treated KD-Control cells. Knockdown of HSP60 induced a significant upregulation (p < 0.05) in SR-A1 mRNA levels that further increased significantly on treatment with higher dose of OxLDL (p < 0.001) (Fig. 8e). Also, a non-significant increment in CD36 mRNA was noted in OxLDL treated KD-Control cells. HSP60 KD cells recorded significant upregulation (p < 0.001) of CD36 on exposure to OxLDL (Fig. 8f). A significant upregulation (p < 0.001) in SR-B1 mRNA expression was recorded following OxLDL exposure to KD-Control cells. However, HSP60 KD cells did record significant changes in SR-B1 mRNA levels in HSP60 KD cells compared to KD-Control (Fig. 8g). It is interesting to note that in conditions of HSP60 downregulation, treatment with OxLDL aggravated levels of SR-A1 and CD36 compared to that in KD-Control cells. Also, OxLDL mediated upregulation of SR-B1 was reduced in HSP60 KD compared to that in KD-Control cells (Fig. 8h).

### HSP60 downregulation enhances OxLDL induced inflammatory phenotype in TDMs

**Figure 9:**
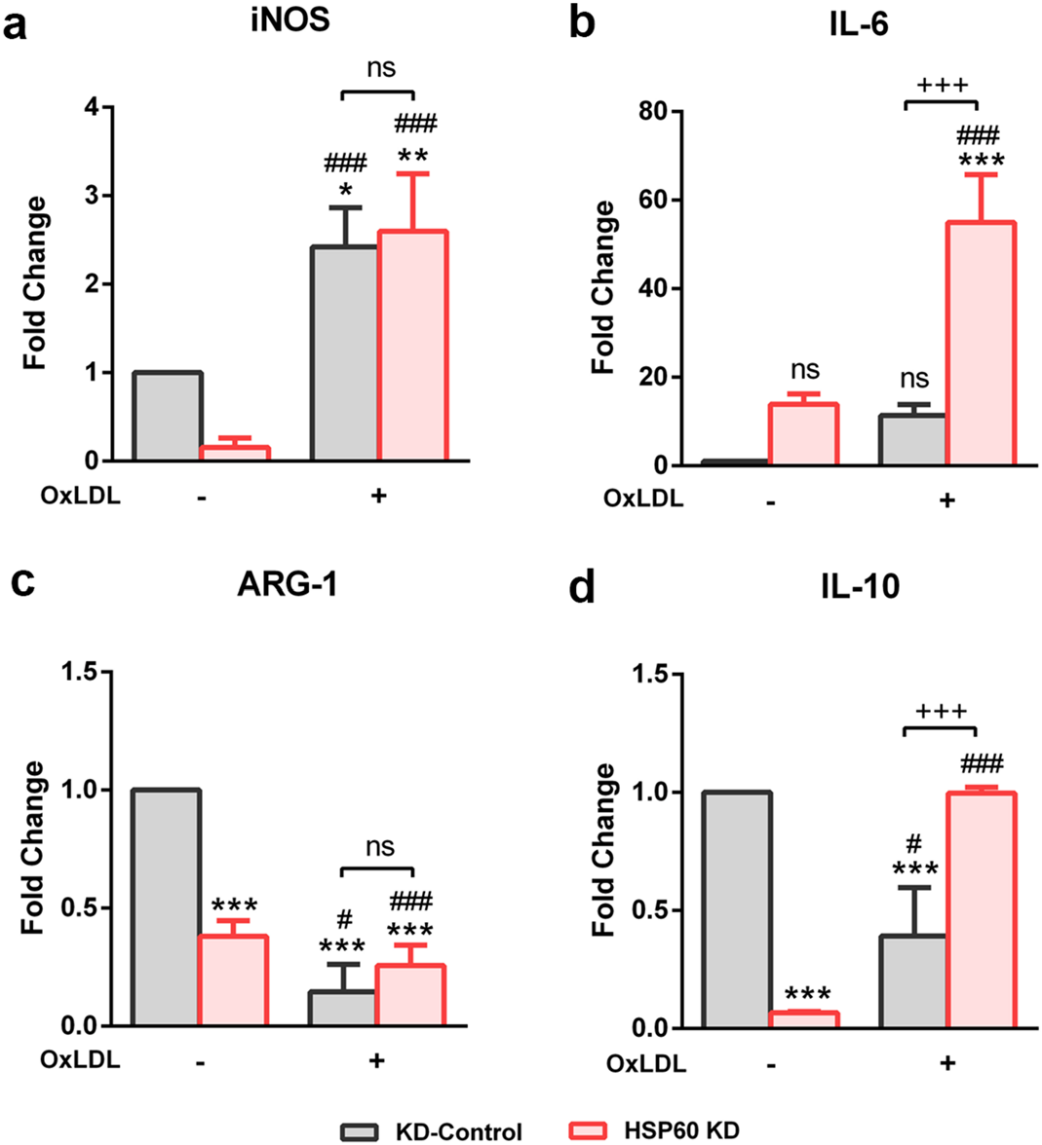
OxLDL induced polarization of HSP60 KD TDMs. OxLDL induced polarization events were checked by assessing the mRNA expression of M1 markers (**a**) iNOS and (**b**) IL-6 and M2 markers (**c**) ARG-1 and (**d**) IL-10 in OxLDL treated KD-Control and HSP60 KD TDMs. Data were expressed as Mean ± SEM (n = 3). *p < 0.05, **p < 0.01, ***p < 0.001 vs untreated KD-Control. ^###^p < 0.001 vs untreated HSP60 KD. ^+^p < 0.05, ^+++^p < 0.001 KD-Control group vs HSP60 KD group, *ns* non-significant, Two-way ANOVA followed by Tukey’s multiple comparison test.

Accumulation of oxidized lipids in macrophages is known to influence the polarization of macrophages^27^ that was studied herein, by assessing the mRNA expression of M1 (iNOS and IL-6) and M2 (ARG-1 and IL-10) macrophage markers. The results showed that OxLDL induced a significant increment (p < 0.05) in iNOS mRNA levels compared to KD-Control. HSP60 KD decreased iNOS expression (non-significant) which was significantly increased (p < 0.001) on exposure to OxLDL (Fig. 9a). In addition, mRNA levels of inflammatory cytokine (IL-6) recorded a moderate upregulation (non-significant) on treatment with OxLDL compared to KD-Control. However, downregulation of HSP60 caused a similar increase (non-significant) in IL-6 mRNA expression whereas, OxLDL treatment further increased the expression by ~ 41.5-fold (p < 0.001) in these cells (Fig. 9b). mRNA levels of ARG-1 decreased significantly (p < 0.001) in OxLDL treated cells compared to KD-Control. Also, HSP60 KD led to a significant decrement (p < 0.001) in ARG-1 mRNA expression as compared to KD-Control. But OxLDL treatment to HSP60 KD cells did not record a significant change in ARG-1 levels (Fig. 9c). Similarly, the mRNA expression of anti-inflammatory cytokine IL-10 was reduced (p < 0.001) in response to OxLDL compared to KD-Control. Also, HSP60 KD significantly decreased IL-10 mRNA levels (p < 0.001) as compared to KD-Control, however, OxLDL treatment in these cells restored the basal levels of IL-10 (Fig. 9d).

## Discussion

Experimental stressors such as lipopolysaccharide (LPS)^16,17^, pro-inflammatory cytokines^17^, fluid shear stress^13^, hypertension^14^, cigarette smoke extract^15^ and high fat diet^25^ induced changes in vascular endothelium and HSP60 are widely reported. Immunologic hypothesis of atherosclerosis strongly correlates with these atherogenic risk factors^13–17,25,28^. In the present study, the regulatory role of HSP60 in atherogenic transformation of vascular endothelium is investigated to establish the missing link between HFHF diet mediated vascular remodelling and endothelial dysfunction. OxLDL treated HUVEC is an established model for studying atherogenic changes but, the role of HSP60 lacks clarity. A preliminary study had demonstrated co-expression of HSP60 and adhesion molecules in human arterial endothelial cells (HAEC) under the influence of cytokines and LPS exposure^17^. OxLDL mediated upregulation of adhesion molecules is also reported in HAEC^17^, HUVEC^29^ and human coronary artery endothelial cells (HCAEC)^30^. These pointers adequately underline the importance of HSP60 chaperone and hence call for a profound investigation. Prima facie evidence from other studies have shown HSP60 expression in early arterial lesions of humans that also support our hypothesis^12,31^. In our study, the observed HSP60 upregulation following OxLDL treatment in HUVEC forms the basis of our further investigation on its modulations during atherogenic arterial remodelling and endothelial dysfunction.

Feeding of high fat diet to C57BL/6J mice is known to enhance body weight gain, induce metabolic perturbations in serum lipid profile and manifest atherogenic changes^32^ that were recorded in our study in the same strain of mice fed with HFHF diet. Hypertrophic lumen dilation and elastin derangement recorded in the thoracic aorta are indicative of pro-atherogenic remodelling. Further, elastin fragmentation and deposition of collagen content in thoracic aorta of HFHF fed mice accounted for a higher collagen-to-elastin ratio. These conditions often culminate in arterial stiffening indicating compromised vascular distensibility and function^33^. At this stage, elevated expression of HSP60 was recorded in the vessel wall pointing towards the role of HSP60 in early stages of atherogenic development. The observed intimal HSP60 staining is in agreement with the previous reports on HSP60 upregulation in tunica intima of early arterial lesions in humans that implies towards recruitment of HSP60-specific mononuclear cells^12^. Further, the upregulation of HSP60 in tunica media perhaps correlates with its expression in VSMCs as the same has also been reported in human atherogenic lesions^31^.

HSP60 surface localization is now an acknowledged atherogenic alteration in EC that is responsible for eliciting an auto-immune response preceding formation of atherosclerotic lesion^14,15^. Overexpression of HSP60 and its surface localization in HUVEC provide compelling evidence that an intracellular HSP60 build-up acts as a preliminary trigger for its translocation to the plasma membrane culminating in its subsequent secretion. Pro-atherogenic action of soluble HSP60 is well known with events such as activation of immune cells^19,20,34^ and VSMC proliferation^35^. Therefore, the said changes in our study are assumed to be the key contributors for the overall thickening of the vessel wall.

The functional changes in vasculature during pro-atherogenic arterial remodelling are predominantly attributed to endothelial dysfunction, which is also an early detectable change during atherogenesis. Depending on the blood flow and luminal shear stress, eNOS catalyses the production of NO in EC that diffuses into the sub-intimal layers and activate VSMCs to regulate vasodilation^33^. The decrement in eNOS mRNA levels observed in HFHF mice supports our claim of endothelial dysfunction. Other studies on high fat diet induced obesity models had shown decreased vascular reactivity indicating endothelial dysfunction^32,36^. Herein, the possible association of HSP60 with endothelial dysfunction was assessed by overexpressing (HSP60-GFP) or silencing (HSP60 KD) HSP60 in HUVEC. Importance of HSP60 upregulation in endothelial dysfunction is emphasized due to the recorded decrement in NO production and eNOS mRNA in HSP60 overexpressed HUVEC; a response that is comparable to OxLDL treated HUVEC. Further, downregulation of HSP60 prevented OxLDL induced eNOS downregulation and NO production thus, implying towards role of endogenous HSP60 as a regulator of eNOS expression.

Endothelial dysfunction further leads to its subsequent activation wherein, upregulation of macrophage chemoattractant protein (MCP-1) and adhesion molecules (VCAM-1 and ICAM-1), facilitates the recruitment of monocytes into the sub-intimal space^37^. In our study, accumulation of macrophages (CD68^+^ area) was observed in thoracic aorta of HFHF fed mice that is explained by the simultaneous upregulation of MCP-1, VCAM-1 and ICAM-1 mRNA levels. These results confirm atherogenic changes in endothelium that also marks the initiation of atherosclerotic lesion formation in keeping with reports of other groups^38,39^. These results provide inconclusive evidence on the possible correlation between HSP60 modulations and subsequent alterations in expression of adhesion molecules. Hence, further evidence in this regard, was generated by inducing HSP60 overexpression (HSP60-GFP) and knockdown (HSP60 KD) in HUVEC. Decrease in OxLDL induced monocyte adhesion following HSP60 knockdown in HUVEC implies to its dependency on HSP60 for EC activation. Also, the HSP60 KD cells failed to elicit transcriptional activation of ICAM-1 following OxLDL treatment in HUVEC, whereas VCAM-1 elicited a similar but weaker response. It can be said that alterations in HSP60 directly impact ICAM-1 expression but VCAM-1 appears to be independent of the HSP60 levels. MCP-1 upregulation in thoracic intimal cells is the hallmark of macrophage recruitment and atherogenic progression^1^. The observed significant decrement of MCP-1 mRNA in HSP60 KD cells following OxLDL treatment is also a key finding that underlines the importance of HSP60 protein in governing the synchrony of atherogenic markers essential for monocyte infiltration. The endothelial expression of cell adhesion molecules in response to OxLDL is known to be regulated by transcription factor NF-κB^1,40,41^. Since HSP60 mediated regulation of NF-κB activation has been documented in cancer cells^21^, it is hypothesized to be operating by a similar mechanism in our study.

Monocyte recruitment in the sub-endothelial space is followed by its differentiation into macrophages that internalize OxLDL to form lipid laden foam cells^42^. In our study, OxLDL induced HSP60 upregulation was observed in TDMs that is in agreement with reports of Frostegard et al*.*^26^. Hyperglycemic stress mediated release of HSP60 from THP-1 cells has been reported by other research group^43^ and the same was recorded by us in OxLDL treated TDMs. This finding is of significance because HSP60 secretion along with other inflammatory cytokines are instrumental in eliciting an immune response that is an essential primary component for atherosclerotic inflammation^19,43^.

Interactions of modified LDL with SRs have been correlated with modulations in their expression coupled with the SR mediated OxLDL uptake leading to foam cell formation^44^. In our study, HSP60 KD in TDMs accounted for significantly higher OxLDL uptake indicating towards an underlying change in SR expression. Despite the high degree of homology, CD36 and SR-B1 have been reported to be distinctly different in their roles in lipid metabolism and atherosclerosis wherein, CD36 plays a major role in contributing to 60–70% uptake and clearance of modified LDL^45^. SR-B1 is highly expressed in liver and macrophages where it mediates cholesterol transfer from cells to HDL for elimination through bile and faeces^46^. Besides CD36, a major share of OxLDL uptake in macrophages have also been attributed to SR-A1. In our study, a comparison of status of SRs observed in OxLDL treated KD-Control TDMs reveals that SR-B1 contributes majorly thus, accounting for less intracellular accumulation. It is interesting to note that higher SR-A1 and CD36 mRNA levels along with lower SR-B1 mRNA in OxLDL treated HSP60 KD TDMs had possibly contributed towards skewed uptake-to-efflux balance resulting in enhanced OxLDL accumulation. These results highlight the regulatory role of HSP60 in recruiting SRs in atherogenic sojourn and macrophage dynamics wherein, significance of basal level of HSP60 in maintaining normal macrophages function is adequately emphasized.

Differentiating macrophages acquire specialized phenotypes in response to the local environment causing macrophage polarization wherein, classically activated M1 and alternatively activated M2 macrophages can be seen^47^. M1 macrophages secrete inflammatory cytokines such as IL-6 and release vasoactive molecules such as nitric oxide, endothelins and eicosenoids^48^. Also, arginase-1 (ARG-1) competitively inhibits iNOS, thus cutting into the pro-inflammatory component of M1 macrophages^49^. In our study, OxLDL induced significant increment in mRNA levels of M1 macrophage markers (iNOS and IL-6) and a decrement in ARG-1 mRNA levels in HSP60 KD cells. However, these cells also recorded a significant increment in the M2 marker (IL-10) but, its relevance in atherogenic progression is not clear. Both pro- and anti-atherosclerotic actions of M2 macrophages have been reported^42,47,50^. Hence, our observations on M1 and M2 macrophage markers imply towards a regulatory role of HSP60 wherein, HSP60 deficient macrophages accounted for polarization towards M1 type that requires further scrutiny.

Our study provides an evidence on OxLDL/HFHF mediated upregulation of HSP60 along with its surface localization and secretion in endothelium. Valuable insights on the role of HSP60 in early pro-atherogenic events were obtained wherein HSP60 upregulation was identified as a crucial trigger for initiating atherogenic changes in vascular endothelium. Besides, the HSP60 secretion synchronized with SR expression and OxLDL uptake. Of note, downregulation of HSP60 in macrophages led to a marked increment in OxLDL accumulation highlighting the significant contribution of basal HSP60 levels in regulating macrophage function of clearing OxLDL. Polarization towards pro-inflammatory subtype was also observed to be augmented in macrophages with lowered HSP60 levels and enhanced OxLDL accumulation. Taken together, it can be tentatively surmised that HSP60 plays differential roles in atherogenesis wherein, its upregulation in ECs is a crucial event that initiates dysfunction and subsequent macrophage recruitment but, similar response in macrophages possibly checks OxLDL accumulation by regulating the synchrony of SRs. Our observations point towards novel cell-type specific functions of HSP60, that are critical with respect to atherosclerosis. These findings are also of relevance in understanding the overlapping sequence of events and role of HSP60 chaperone during atherogenic initiation.

## Materials and methods

### Animal experiments

The animal experimental protocol (MSU-Z/IAEC/2/09-2017) was approved by the Institutional Animal Ethical Committee (IAEC), Department of Zoology, The Maharaja Sayajirao University of Baroda, Vadodara, Gujarat, India and the experiments were performed in accordance with the guidelines of Committee for the Purpose of Control and Supervision of Experiments on Animals (CPCSEA; 827/GO/Re/S/04/CPCSEA). C57BL/6J male mice (20–22 g) of age 4–6 weeks were obtained from Advanced Center for Treatment, Research and Education in Cancer (ACTREC), Navi Mumbai, India. Animals were housed as 3–5 per cage and fed ad libitum with standard laboratory chow and water in rooms maintained in 12 h light/12 h dark cycle at 23 °C to 25 °C with humidity between 50 and 70%. After 10-days acclimatization period, they were randomly divided into the Control group (n = 6, fed with standard chow) and the HFHF group (n = 10, fed with HFD containing 35.3% fat content and 20% fructose in water). Food intake and body weights were recorded every alternate day. After 16 weeks, blood was collected via retro-orbital sinus after overnight fasting, animals were euthanized and thoracic aortas were excised, fixed in 4% buffered paraformaldehyde (PFA) and embedded in paraffin using standard protocol for histochemical analysis. For gene expression studies, the samples were snap frozen in liquid nitrogen and stored at − 80 °C until use. Serum was isolated from blood using standard protocol and was subjected to lipid profile analysis using commercially available kits (Reckon Diagnostic kits, Vadodara, Gujarat, India).

### Histology and morphometric analysis

Tissues fixed in 4% PFA (pH 7.2) were dehydrated in a graded series of ethanol and embedded in paraffin blocks. 5 µm serial sections were cut on microtome and stained with hematoxylin–eosin (H × E; Sigma-Aldrich, USA). The sections were observed and photographed using Leica DMRB microscope (Leica Microsystems, Germany). The intima-media thickness (IMT) and lumen area were measured using ImageJ software (NIH, Bethesda, USA) by an investigator blinded to group assignment.

### Elastin autofluorescence and collagen staining

Elastin autofluorescence was recorded from the H × E stained sections using FLoid imaging station (Thermo Fisher Scientific, USA)^51^. Elastin breaks, defined as discontinuity of an elastic fiber with boundaries at both sides clearly visible, were counted from the images. For collagen staining, paraffin-embedded sections were de-paraffinized with xylene and rehydrated by immersion in a graded series of ethanol. The sections were then stained with 0.1% direct red 80 (Sigma Aldrich, USA) in saturated aqueous solution of picric acid for 1 h at room temperature (RT) and observed under Leica DMRB microscope (Leica Microsystems, Germany). Images were captured and quantitative analysis of the images for collagen and elastin content was done using ImageJ software (NIH, Bethesda, USA)^52,53^. Aortic stiffness was calculated as ratio of collagen to elastin.

### Immunohistochemical analysis

5 µm thick sections of thoracic aortas were subjected to immunohistochemical staining for detection of HSP60 or CD68. Briefly, paraffin embedded sections were de-paraffinized in xylene, re-hydrated in graded series of ethanol and washed with phosphate buffered saline (PBS). Antigen retrieval was carried out in sodium citrate buffer at 95 °C for 20 min and endogenous peroxidases were masked with 3% hydrogen peroxide for 20 min in dark. Sections were blocked in 1% fetal bovine serum (FBS) for 30 min at RT and incubated overnight with primary antibodies (HSP60 at 1:200; Cell Signaling Technology, USA and CD68 at 1:100; Dako, Agilant, USA) at 4 °C in humidified chamber. After washing with PBS, sections were incubated with horseradish peroxidase (HRP) conjugated secondary antibody (Dako, Agilant, USA) for 1 h at RT. Thereafter, sections were washed thoroughly with PBS and DAB substrate (Dako, Agilant, USA) was added followed by counter-staining with haematoxylin. Sections were observed and images were captured under Leica DMRB microscope (Leica Microsystems, Germany). Quantification of positively stained regions was carried out using Fiji software^54^ (ImageJ, NIH, Bethesda, USA).

### Cell culture

HUVEC were purchased from HiMedia Laboratories (Mumbai, India) and maintained in tissue culture flasks coated with 0.5% gelatin in HiEndoXL Endothelial Growth medium (HiMedia Laboratories, Mumbai, India) supplemented with HiEndoXL Endothelial Growth Supplement (HiMedia Laboratories, Mumbai, India) and 1× antibiotic antimycotic solution (HiMedia Laboratories, Mumbai, India). The cultures were maintained at 37 °C and 5% CO_2_. Logarithmically growing cells (passages 2–6) were used for all experiments. Human monocyte (THP-1) cell line was obtained from National Centre for Cell Science (NCCS), Pune, Maharashtra, India and maintained in RPMI-1640 medium (HiMedia Laboratories, Mumbai, India) supplemented with L-glutamine (2 mmol/l), 10% FBS (Gibco, Thermo Fisher Scientific, USA) and 1× antibiotic–antimycotic solution (HiMedia Laboratories, Mumbai, India) in a humidified atmosphere with 5% CO_2_ at 37 °C. For the induction of cell differentiation, THP-1 cells (1.5 × 10^6^ per ml) were seeded in serum-free RPMI-1640 with 50 nM PMA for 24 h. After incubation, non-adherent cells were removed by aspiration, and the adherent THP-1 derived macrophages (TDMs) were washed with PBS before experimental treatments.

### Plasmids and transfection

HUVEC were transfected with either pCMV3-HSPD1-GFPSpark (HSP60-GFP; Sino Biological Inc., China) or pcDNA3.1-Hsp60-MycHis (pcDNA-HSP60) plasmids (kind gift from Dr. Thomas Corydon, Aarhus University^55^) for overexpressing HSP60. Empty vectors were used as control (Vector). For HSP60 knockdown (HSP60 KD), HUVEC and TDMs were transfected with pshRNA-609 containing shRNA sequence against *hspd1* cloned between BamHI and HindIII sites of p*Silencer* 2.0-U6 vector (kind gift from Dr. Thomas Corydon, Aarhus University^55^). p*Silencer* 2.0-U6 Negative Control (Thermo Fisher Scientific, USA) containing scrambled shRNA sequence lacking homology to human genome was used as negative control (KD-Control). Transfection was carried out using lipofectamine 3000 (Thermo Fisher Scientific, USA) according to manufacturer’s instructions with minor modifications. After 4 h of transfection, cells were washed with warm PBS 3–4 times and incubated in respective growth medium for 24 h for HSP60 overexpression group and 48 h for HSP60 KD group. The transfection efficiency was assessed by checking HSP60 mRNA and protein expression. HSP60 KD cells were further exposed to OxLDL (80 µg/ml) in serum-free medium for 24 h (HSP60 KD + OxLDL).

### Cell viability assay

Cell viability was assessed by MTT (3-(4,5-dimethylthiazol-2-yl)-2,5-diphenyltetrazolium bromide) assay. After experimentation, the cells were washed with PBS, followed by addition of 0.5 mg/ml MTT to each well and incubation at 37 °C for 4 h. The purple formazan crystals formed were dissolved in DMSO and the absorbance was recorded at 590 nm using Synergy HTX Multimode Microplate Reader (BioTek Instruments Inc., USA). The results were represented as percentage cell viability with respect to control.

### LDH release assay

The amount of LDH released from the cells was evaluated by using the LDH cytotoxicity detection kit (Takara Bio Inc., Japan) as per the manufacturer’s protocol with minor modifications. Briefly, culture supernatant was collected and cleared of the debris by centrifugation at 800×*g* for 5 min. The clarified supernatant was mixed with equal amount of reaction mixture and incubated in dark for 30 min at RT. The absorbance was recorded at 490 nm using Synergy HTX Multimode Microplate Reader (BioTek Instruments Inc., USA). The percentage of LDH released in the culture media was calculated relative to maximum control samples wherein cells were lysed with 0.1% Triton-X-100 representing 100% LDH release.

### HSP60 secretion

Conditioned media from cells was collected and centrifuged at 800×*g* for 5 min to remove cellular debris. Levels of HSP60 in conditioned media was detected using anti-HSP60 ELISA kit according to manufacturer’s protocol (RayBiotech, USA). The concentration of HSP60 in samples was determined from standard curve using GraphPad Prism 6.0.

### Immunofluorescence staining

Intracellular and surface immunofluorescence staining was carried out as per protocol described by Jakic et al.^14^ with minor modifications. HUVEC were grown on gelatin coated sterile coverslips and subjected to HSP60 overexpression (pcDNA-HSP60) or OxLDL treatment for 24 h. For intracellular staining, cells were washed with warm PBS and fixed with 2% PFA for 10 min at RT followed by 100% methanol (MeOH) for 10 min at RT for intracellular staining. Cells were washed thrice with wash buffer (0.5% BSA in PBS) followed by blocking with 1% BSA in PBS for 1 h at RT. Cells were incubated overnight with HSP60 rabbit monoclonal antibody (1:200; Cell Signaling Technology, USA) at 4 °C in humidified chamber. After washing thrice with wash buffer, cells were incubated with Alexa Fluor-488 anti-rabbit secondary antibody (Invitrogen, Thermo Fisher Scientific, USA) for 1 h in dark at RT. Cells were washed thrice in wash buffer and coverslip was mounted using fluoroshield with DAPI (Sigma-Aldrich, USA).

For surface staining, cells were fixed with 1% PFA for 10 min at RT, washed with washed buffer and blocked with 1% BSA in PBS for 1 h at RT. Cells were incubated overnight with HSP60 rabbit monoclonal antibody at 4 °C in humidified chamber, washed and incubated with Alexa Fluor-488 anti-rabbit secondary antibody for 1 h at RT in dark. Cells were washed thrice with wash buffer and subsequently incubated with β-actin mouse monoclonal antibody (1:100; Invitrogen, Thermo Fisher Scientific, USA) at 4 °C in humidified chamber in dark. After washing thrice, Alexa Fluor-568 anti-mouse secondary antibody (Invitrogen, Thermo Fisher Scientific, USA) was added for 1 h RT in dark. After washing, coverslip was mounted in fluoroshield with DAPI. For negative controls, the primary antibodies were excluded. Immunofluorescent cells were observed and images were captured using Zeiss LSM 710 confocal microscope (Carl Zeiss AG, Germany). Images were analysed using ImageJ (NIH, Bethesda, USA). All negative controls did not show any fluorescence.

### Nitric oxide production

Levels of nitric oxide (NO) produced can be determined by measuring its reduced form (nitrite) using Griess’ reagent^56^. Briefly, cell supernatant was centrifuged to remove debris and clear supernatant was mixed with equal volume of Griess’ reagent (Sigma-Aldrich, USA). The mixture was incubated for 30 min at RT in dark and absorbance was recorded at 540 nm using Synergy HTX multimode reader (BioTek Instruments, USA). The concentration of nitrite was determined from sodium nitrite standard curve.

### OxLDL uptake study

Lipids accumulation in the cells was monitored by staining intracellular lipid granules with oil red O (ORO) stain. Briefly, the cells were fixed in 4% PFA for 45 min, washed with PBS and stained with ORO stain (0.5%) for 30 min at RT. After washing with distilled water, cells were observed under Leica DM750 microscope (Leica Microsystems, Germany). For quantitative analysis, the accumulated ORO stain was extracted in 50% isopropanol and absorbance was recorded at 495 nm using Synergy HTX Multimode Microplate Reader (BioTek Instruments Inc., USA).

### RNA isolation and quantitative RT-PCR

Total RNA was isolated using Trizol reagent (Invitrogen, Thermo Fisher Scientific, USA) according to manufacturer’s protocol. 1 µg of RNA was reverse transcribed to cDNA using iScript cDNA synthesis kit (Bio-Rad Laboratories, USA). The cDNA was subjected to quantitative PCR analysis using Power Up SYBR Green Master Mix (Thermo Fisher Scientific, USA) in QuantStudio-3 Real-Time PCR System (Applied Biosystem, Thermo Fisher Scientific, USA). Primer sequences specific for target mRNAs are mentioned in Table 1. Expression levels were analysed using 2^–∆∆CT^ method relative to GAPDH.

**Table 1:** Primer sequences for quantitative PCR.

Gene name	Forward primer (5′ → 3′)	Reverse primer (5′ → 3′)
**Human**		
*HSP60*	GTTGGGGGACCGCTCATT	CCCGGCCATCCTTATAGACG
*eNOS*	CCTCGTCCCTGTGGAAAGAC	GTGGTCCACGATGGTGACTT
*VCAM-1*	GGGAAGCCGATCACAGTCAA	GGGACTTCCTGTCTGCATCC
*ICAM-1*	GCTGTCTACTGACCCCAACC	GGTGACCTTGAATGTGACATGG
*MCP-1*	TCTGTGCCTGCTGCTCATAG	CTTCTTTGGGACACTTGCTGC
*GAPDH*	GAGTCAACGGATTTGGTC	GACAAGCTTCCCGTTCTC
*SR-B1*	GAAGGCATCCCCACCTATCG	AATTCCAGACTCCAGGCACG
*SR-A1*	AAAGTTCGACTGGTCGGTGG	CCCAAGCTCCTACAGACGAC
*CD36*	CGAGGAAGCCACTTTGGTGA	TGGTTTCTACAAGCTCTGGTTCT
*iNOS*	CGCATGACCTTGGTGTTTGG	CATAGACCTTGGGCTTGCCA
*IL-6*	ACCCCCAGGAGAAGATTCCA	GATGCCGTCGAGGATGTACC
*ARG-1*	GGGTTGACTGACTGGAGAGC	CGTGGCTGTCCCTTTGAGAA
*IL-10*	AGCTCCAAGAGAAAGGCATCT	TCGCCACCCTGATGTCTCA
**Mice**		
*HSP60*	TGATGTTGGCTGTGGATGCT	GACACCCTTTCTTCCAACCTTT
*eNOS*	TGGAAGGGAAGTGCAGCAAA	GGCCAGTCTCAGAGCCATAC
*VCAM-1*	CTGGGAAGCTGGAACGAAGT	GCCAAACACTTGACCGTGAC
*ICAM-1*	GTGGGTCGAAGGTGGTTCTT	AAACAGGAACTTTCCCGCCA
*MCP-1*	TGACCCCAAGAAGGAATGGG	GACCTTAGGGCAGATGCAGTT
*GAPDH*	GTCGGTGTGAACGGATTTGG	AGATGCCTGCTTCCCATTCT

### Immunoblotting

For extracting total protein lysate, tissue was homogenized in RIPA buffer (50 mM tris (pH 8.0), 150 mM NaCl, 0.5% sodium deoxycholate, 0.1% sodium dodecyl sulfate, 1% triton-X-100) containing protease inhibitor cocktail (Sigma Aldrich, USA) and 1 mM PMSF, followed by incubation at 4 °C for 2 h. Cell pellet was lysed in RIPA buffer for 30 min at 4 °C. The lysate were centrifuged at 10,000 rpm at 4 °C for 20 min and resultant supernatant was subjected to protein estimation using Bio-Rad protein assay dye reagent (Bio-Rad Laboratories, USA). 25 µg of protein from each sample was fractionated by SDS-PAGE and transferred onto PVDF membrane using standard protocol of Trans-Blot Turbo Transfer System (Bio-Rad Laboratories, USA). Membrane was blocked using 3% BSA in Tris buffered saline (TBS) for 1 h at RT, followed by overnight incubation in anti-HSP60 (1:750; Cell Signaling Technology, USA) and anti-β-actin (1:1000; Invitrogen, Thermo Fisher Scientific, USA) at 4 °C. Thereafter, the membrane was washed with TBST and probed with HRP-labelled anti-rabbit secondary antibody (1:2000; Cell Signaling Technology, USA) for 90 min at RT. Expression of the immune-reactive proteins was detected using Clarity Western ECL Substrate (Bio-Rad Laboratories, USA) according to the instruction manual and chemiluminiscence was recorded using iBright CL1000 Imaging System (Invitrogen, Thermo Fisher Scientific, USA).

### Monocyte endothelial cell adhesion assay

Monocyte endothelial cell adhesion assay was carried out as described by Yanaka et al*.*^57^ with minor modifications. HUVEC were seeded in 12 well plate and allowed to reach ~ 80% confluence. Then, cells were transfected as described earlier or treated with OxLDL (80 µg/ml) for 24 h. THP-1 cells were labelled with Hoechst 33342 (1 µg/ml; HiMedia Laboratories, India) for 15 min in incomplete RPMI-1640 in dark. 0.5 × 10^5^ THP-1 cells labelled with Hoechst stain were added to the wells containing HUVEC and incubated for 30 min. Unbound THP-1 cells were removed by washing thrice with warm PBS. Bound cells were observed and images were captured using FLoid Cell imaging station (Thermo Fisher Scientific, USA). Number of HUVEC and THP-1 cells were counted and % monocyte adhesion was calculated relative to ECs.

### Statistical analysis

All the statistical analysis was carried out using GraphPad Prism 6.0. Differences between groups were compared by unpaired two-tailed Student’s t-test (for comparison between two groups) and one-way ANOVA followed by Tukey’s test (for comparisons between three or more groups). For analysis of data containing two independent variables, differences were compared using Two-way ANOVA followed by Tukey’s test. Results were expressed as mean ± S.E.M. For all analysis, differences were considered statistically significant at *p ≤ 0.05, **p ≤ 0.01 and ***p ≤ 0.001.

## Supplementary Information

Supplementary Information.
