# Supplementary material for: HSP60 knockdown exerts differential response in endothelial cells and monocyte derived macrophages during atherogenic transformation
Source: Sci Rep. 2021 Jan 13;11:1086. doi: 10.1038/s41598-020-79927-2 (PMC7807046; doi:10.1038/s41598-020-79927-2)
Supplement: Supplementary file 1 — Supplementary Information. [file 41598_2020_79927_MOESM1_ESM.pdf]

SUPPLEMENTAL MATERIAL

HSP60 knockdown exerts differential response in endothelial cells and monocyte derived macrophages during atherogenic transformation

Kavita Shirsath[1], Apeksha Joshi[1], Aliasgar Vohra[1], Ranjitsinh Devkar[1]*

[1] Department of Zoology, Faculty of Science, The Maharaja Sayajirao University of Baroda, Vadodara-390002, Gujarat, INDIA.

*Correspondence: rv.devkar-zoo@msubaroda.ac.in

Supplementary Table 1: Calorie contents of the experimental diets

**Feed type**	**Energy content (Kcal/kg)**	**Energy intake via food (Kcal/day/mice)**
Chow	3090	8.11±0.78
High fat diet	5000	9.22 ±0.75*

*P<0.05 vs Chow, unpaired two-tailed Student's t-test (n=6).

Supplemental Figures

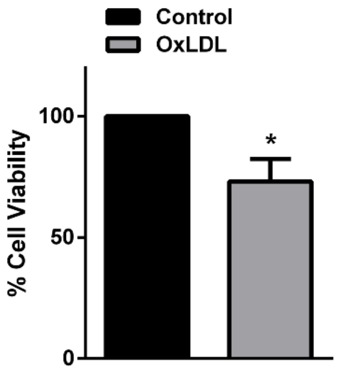

Supplemental Figure S1: Effect of OxLDL on cell viability in HUVEC. Cells were treated with OxLDL (80 µg/ml) for 24 h and the cell viability was determined by MTT assay. % cell viability was calculated relative to control. Data were represented as Mean ± SEM (n=3). *p<0.05, unpaired two-tailed Student's t-test.

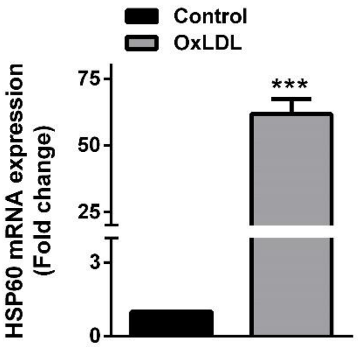

Supplemental Figure S2: Effect of OxLDL on HSP60 mRNA expression. HUVEC were treated with OxLDL (80 µg/ml) for 24 h and HSP60 mRNA expression was assessed by quantitative RT-PCR. Data were represented as Mean ± SEM (n=3). ***p<0.001, unpaired two-tailed Student's t-test.

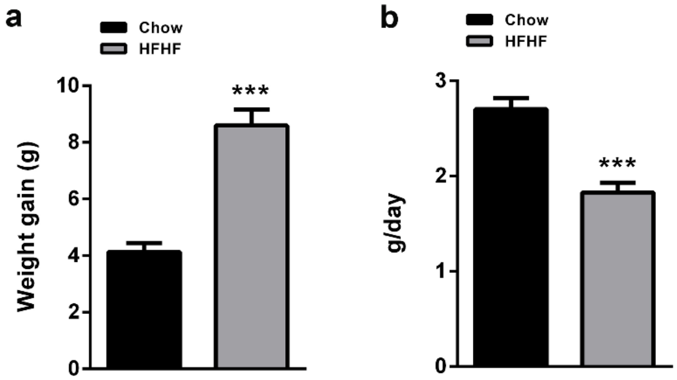

Supplemental Figure S3: Weight gain and food intake in HFHF diet fed mice. The graphs represent (a) final weight gain and (b) average food intake per day in chow diet and HFHF diet fed mice. Data were represented as Mean ± SEM (n=6). ***p<0.001, unpaired two-tailed Student's t-test.

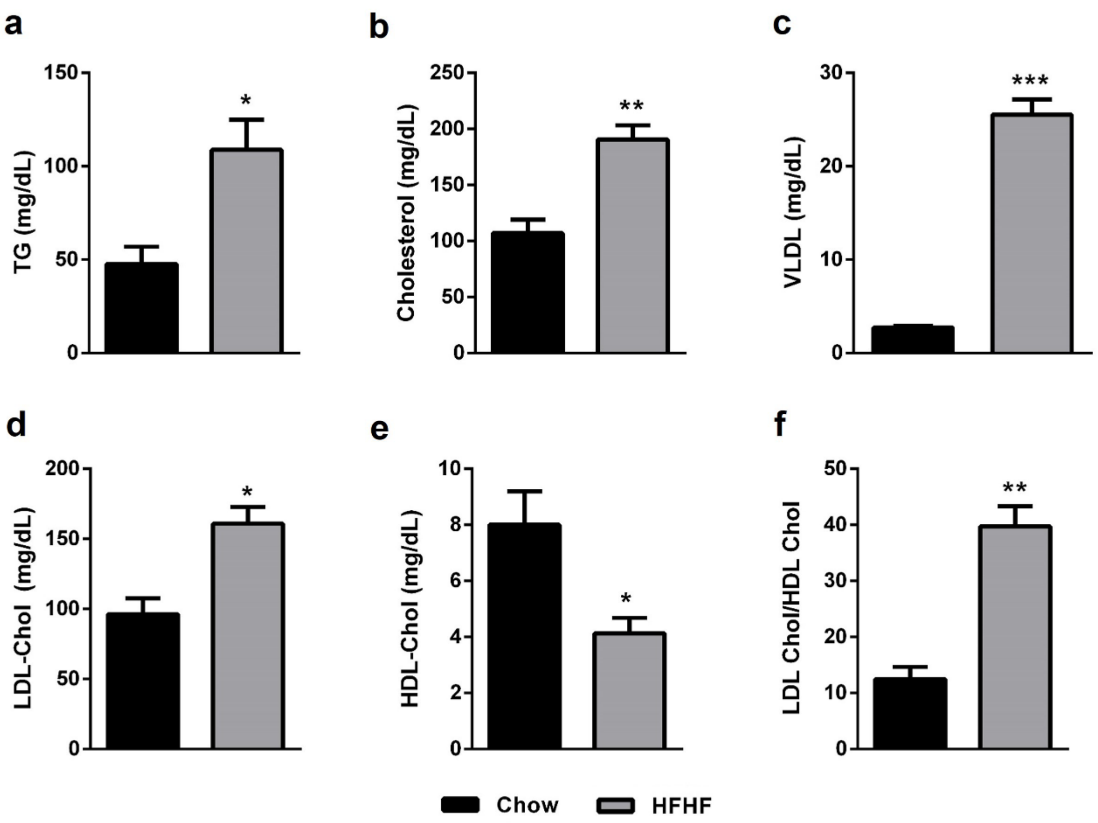

Supplemental Figure S4: Serum lipid profile of HFHF diet fed mice. The graphs represent serum titers of (a) triglycerides (TG), (b) total cholesterol (TC), (c) very low density lipoprotein (VLDL), (d) low density lipoprotein cholesterol (LDL-Chol), (e) high density lipoprotein cholesterol (HDL-Chol) and (f) LDL-Chol/HDL-Chol ratio were assayed (n=6). Data were represented as Mean ± SEM. *p<0.05, **p<0.01, ***p<0.001, ns- non-significant, unpaired two-tailed Student's t-test.

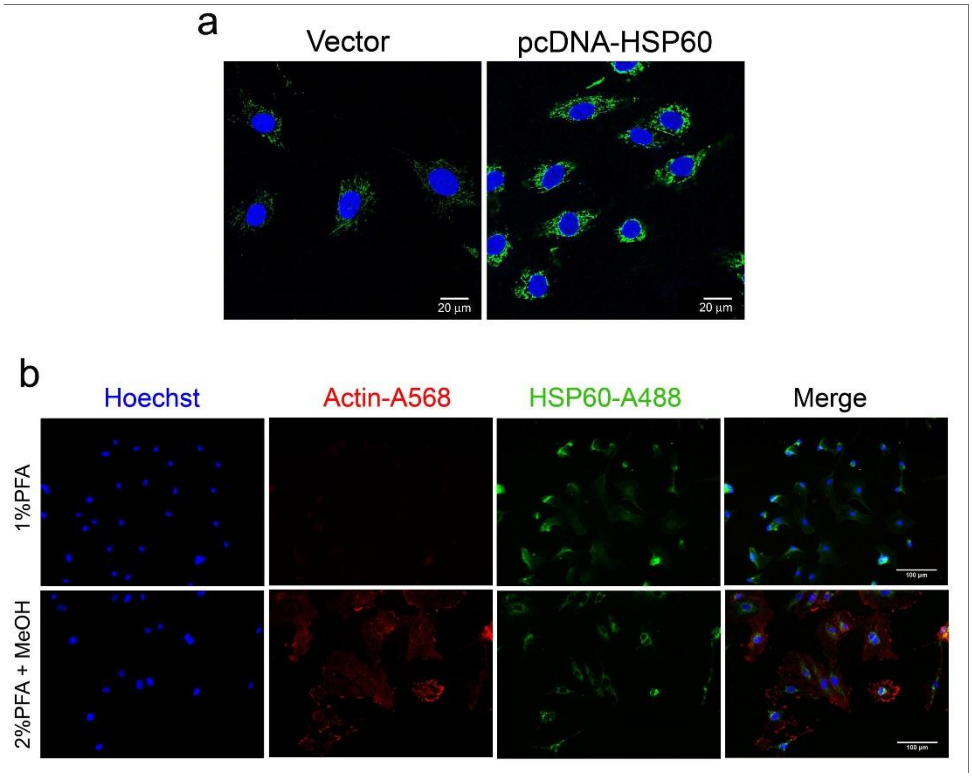

Supplemental Figure S5: HSP60 overexpression and standardization of surface staining protocol in HUVEC. (a) Cells were transfected with pcDNA3.1-Hsp60-MycHis (pcDNA-HSP60) or empty vector (Vector) and the overexpression was checked by intracellular immunostaining of Vector control and pcDNA-HSP60 cells after 24 h of transfection. Scale bar= 20 µm. (b) OxLDL treated HUVEC were subjected to fixation with 1% PFA (for surface staining) or 2% PFA followed by permeabilization with methanol (2% PFA + MeOH; for intracellular staining). Representative images of nuclei (Hoechst), β-actin (A568) and HSP60 (A488) are shown. Scale bar= 100 µm.

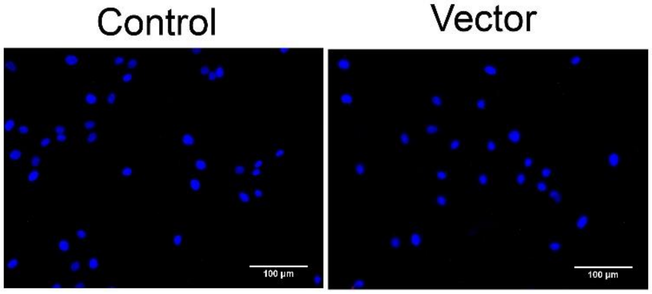

Supplemental Figure S6: Surface localization of HSP60 in Vector control cells. HUVEC transfected with pcDNA3.1-MycHis empty plasmid (Vector) were surface immunostained for HSP60 by following 1%PFA fixation protocol. Non-transfected cells were used as Control. Both the groups were observed to be negative for HSP60 (A488) and β-actin (A568) with Hoechst stained nuclei. Scale bar=100 µm.

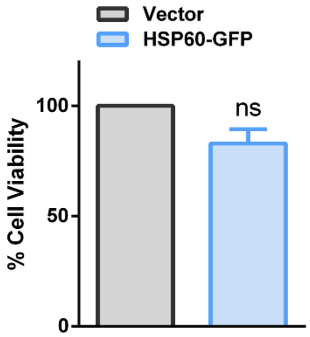

Supplemental Figure S7: Cell viability of HSP60-GFP HUVEC. Cell viability of HUVEC overexpressing HSP60 (HSP60-GFP) was determined by MTT assay. % cell viability was calculated relative to Vector control. Data represents Mean ± SEM (n=3). ns- non-significant, unpaired two-tailed Student's t-test.

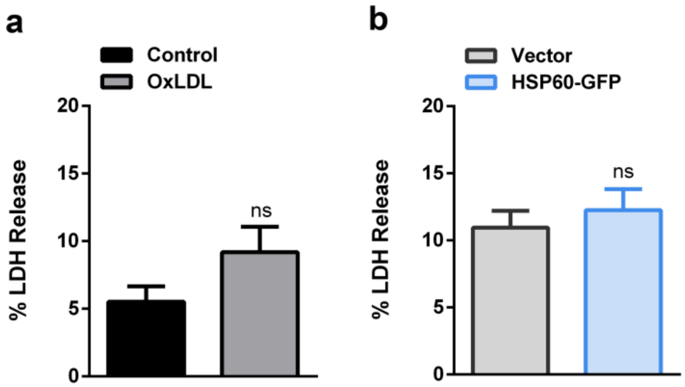

Supplemental Figure S8: LDH release from HUVEC. Cells were (a) treated with OxLDL and (b) transfected with HSP60-GFP for 24 h and the percentage of LDH release was determined relative to maximum control. Data were represented as Mean ±SEM (n=3). ns- non-significant, unpaired two-tailed Student's t-test.

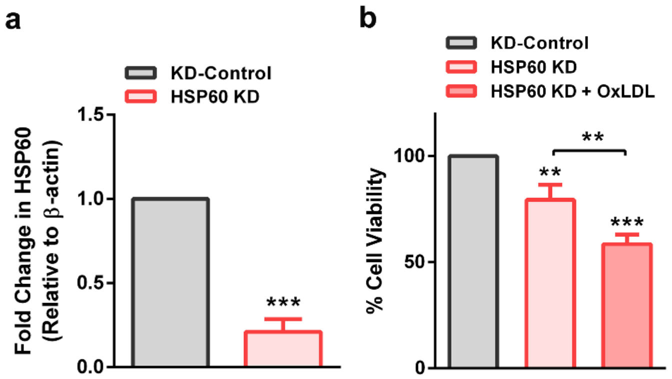

Supplemental Figure S9: Cell viability in HSP60 KD HUVECs. Cell were transfected with pshRNA-609 and (a) densitometry of immunoblot (n=4) confirmed HSP60 knockdown (HSP60 KD). ***P<0.001 vs KD-Control, unpaired two-tailed Student's t-test. (b) HSP60 KD HUVEC were treated with OxLDL (80 µg/ml) for 24 h and cell viability was determined by MTT assay (n=3). % cell viability was calculated relative to KD-Control. Data were represented as Mean ± SEM. **p<0.01, ***p<0.001 vs respective untreated control, One way-ANOVA followed by Tukey's multiple comparison test**.**

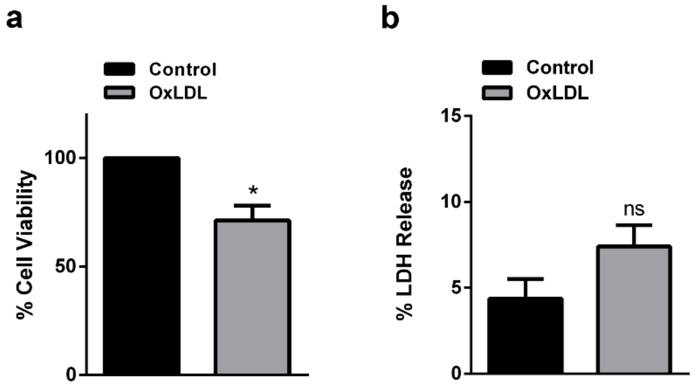

Supplemental Figure S10: Cell viability and LDH release in OxLDL treated TDMs. Cells were treated with OxLDL (80 µg/ml) for 24 h and (a) cell viability was determined by MTT assay. % cell viability was calculated relative to control. Further, (b) percentage of LDH release was determined relative to maximum control. Data were represented as Mean ± SEM (n=3). *p<0.05 vs Controls, ns- non-significant, unpaired two-tailed Student's t-test**.**

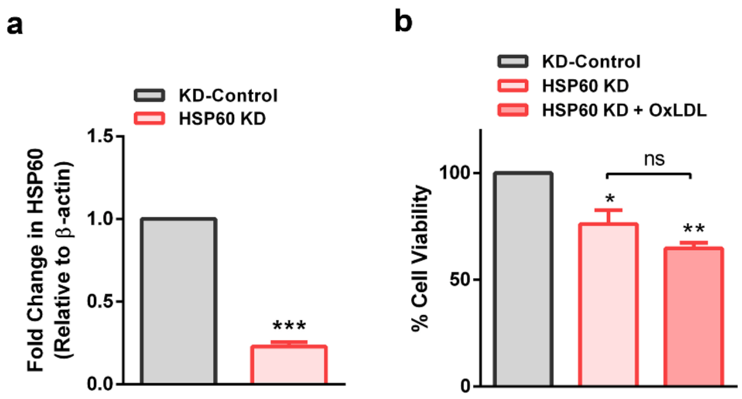

Supplemental Figure S11: Cell viability of HSP60 knockdown TDMs. Cells were transfected with pshRNA-609 and knockdown was confirmed by immunoblotting. (a) Graph represents densitometry of the immunoblot (n=4). ***P<0.001 vs KD-Control, unpaired two-tailed Student's t-test. (b) HSP60 KD TDMs were treated with OxLDL and cell viability was determined by MTT assay. % cell viability was calculated relative to KD-Control (n=3). Data were represented as Mean ± SEM. *p<0.05, **p<0.01 vs KD-Controls, ns- non-significant, One way-ANOVA followed by Tukey's multiple comparison test.

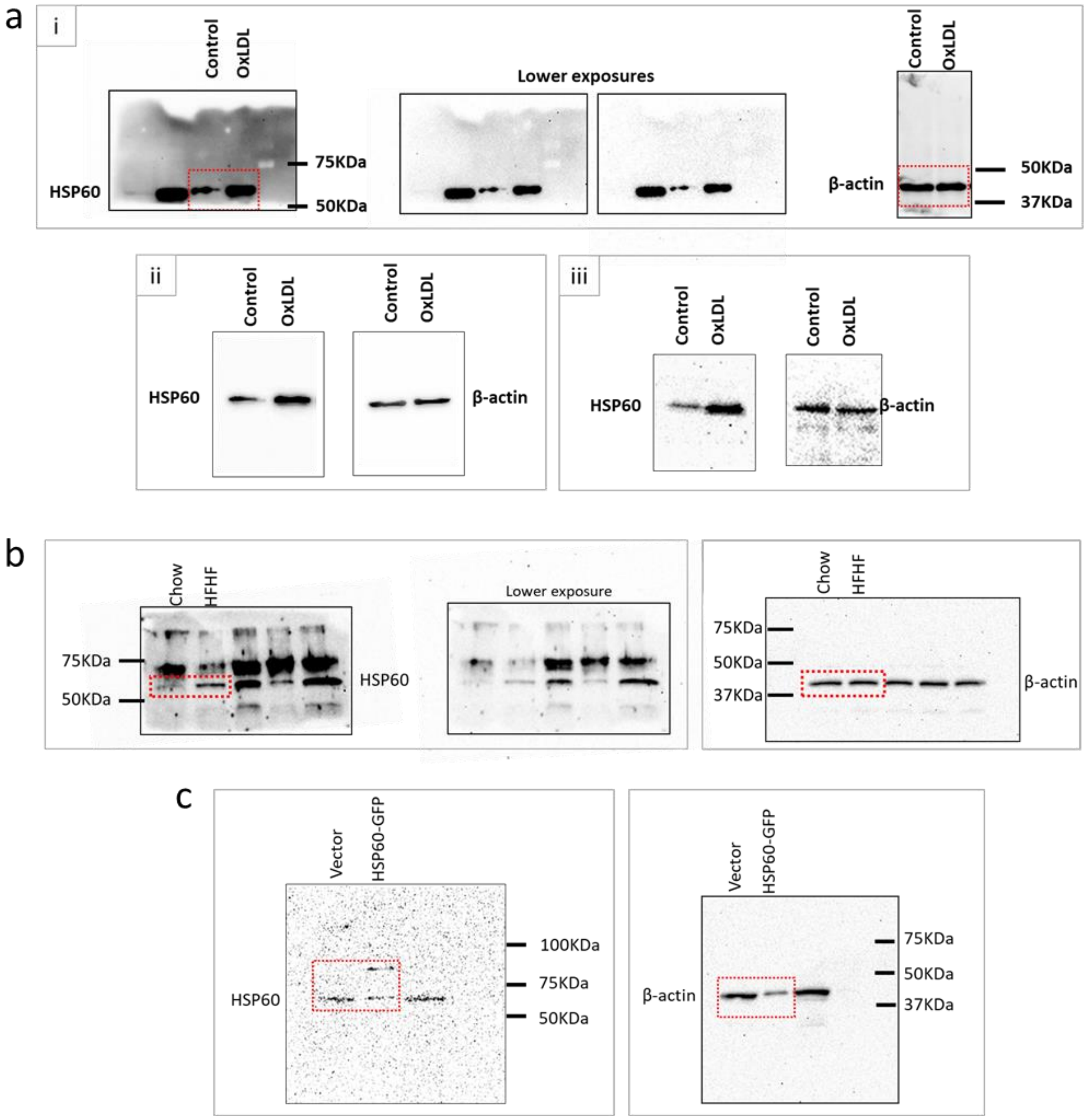

Supplemental Figure S12: Full length images of western blot represented in (a) Figure 1a, (b) Figure 2h and (c) Figure 3d of the manuscript. The HSP60 blot in section (a-i) was cut prior to hybridization with antibody and hence, blots of the experimental replicates are represented in section (ii & iii). The red boxes represent parts of blot displayed in the manuscript. Lower exposures of blots are also presented wherever, high contrast images were used in manuscript.

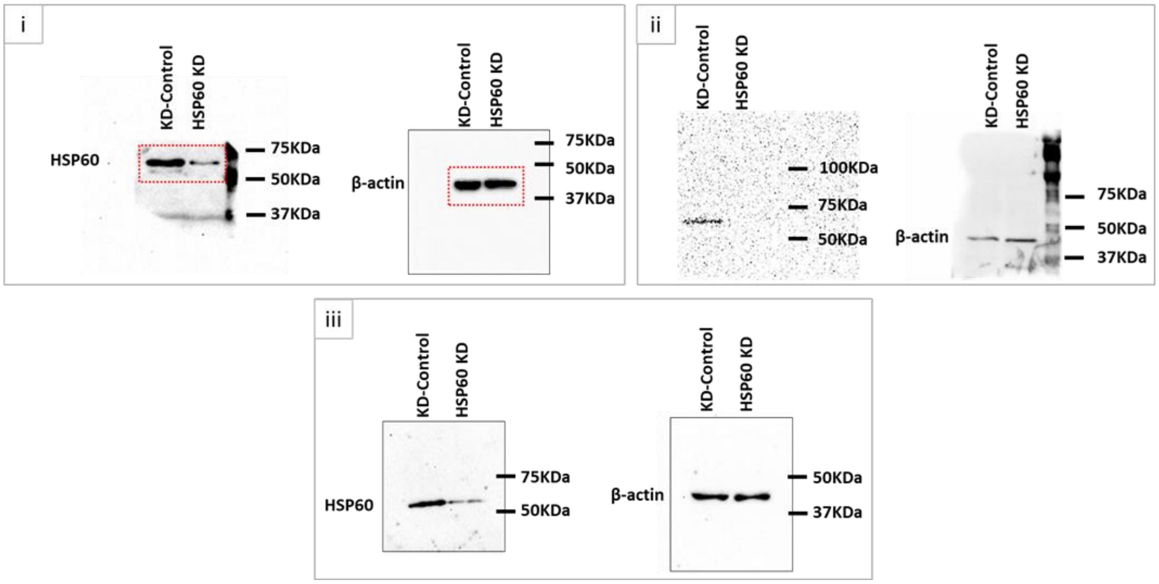

Supplemental Figure S13: Full length images of western blot represented in Figure 5a of the manuscript. The HSP60 blot in section (i) was cut prior to hybridization with antibody and hence, images of blots from experimental replicates are shown in (ii) & (iii). Red boxes represents parts of the blots represented in the manuscript.

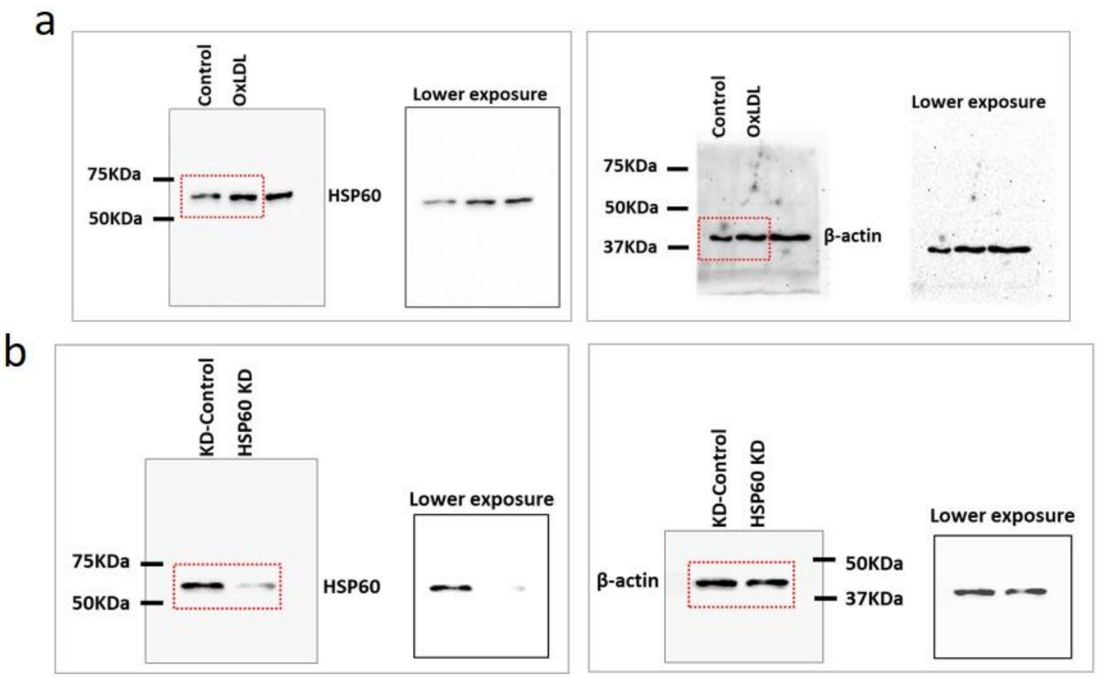

Supplemental Figure S14: Full length images of western blot represented in (a) Figure 7a and (b) Figure 8b of the manuscript. Lower exposure of the blots is shown where high contrast image was included in the manuscript. Red boxes represents parts of the blots represented in the manuscript.
